# High-resolution aCGH and expression profiling identifies a novel genomic subtype of ER negative breast cancer

**DOI:** 10.1186/gb-2007-8-10-r215

**Published:** 2007-10-07

**Authors:** Suet F Chin, Andrew E Teschendorff, John C Marioni, Yanzhong Wang, Nuno L Barbosa-Morais, Natalie P Thorne, Jose L Costa, Sarah E Pinder, Mark A van de Wiel, Andrew R Green, Ian O Ellis, Peggy L Porter, Simon Tavaré, James D Brenton, Bauke Ylstra, Carlos Caldas

**Affiliations:** 1 Breast Cancer Functional Genomics, Cancer Research UK Cambridge Research Institute and Department of Oncology University of Cambridge, Li Ka-Shing Centre, Robinson Way, Cambridge CB2 0RE, UK; 2 Computational Biology Group, Cancer Research UK Cambridge Research Institute and Department of Oncology University of Cambridge, Li Ka-Shing Centre, Robinson Way, Cambridge CB2 0RE, UK; 3 Functional Genomics of Drug Resistance, Cancer Research UK Cambridge Research Institute and Department of Oncology University of Cambridge, Li Ka-Shing Centre, Robinson Way, Cambridge CB2 0RE, UK; 4 Computational Biology Group, Department of Applied Mathematics and Theoretical Physics, University of Cambridge, Centre for Mathematical Sciences, Wilberforce Road, Cambridge CB3 0WA, UK; 5 Histopathology, Nottingham City Hospital NHS Trust and University of Nottingham, Nottingham NG5 1PB, UK; 6 Cambridge Breast Unit, Addenbrookes Hospital, Cambridge University Hospitals NHS Foundation Trust, Hills Road, Cambridge, UK; 7 Department of Pathology, VU University Medical Center, PO Box 7057, 1007MB Amsterdam, The Netherlands; 8 Department of Biostatistics, VU University Medical Center, PO Box 7057, 1007MB Amsterdam, The Netherlands; 9 Department of Mathematics, Vrije Universiteit, Amsterdam, Netherlands; 10 Division of Human Biology, Division of Public Health Sciences, Fred Hutchinson Cancer Research Center, Seattle, WA 98109, USA

## Abstract

**Background:**

The characterization of copy number alteration patterns in breast cancer requires high-resolution genome-wide profiling of a large panel of tumor specimens. To date, most genome-wide array comparative genomic hybridization studies have used tumor panels of relatively large tumor size and high Nottingham Prognostic Index (NPI) that are not as representative of breast cancer demographics.

**Results:**

We performed an oligo-array-based high-resolution analysis of copy number alterations in 171 primary breast tumors of relatively small size and low NPI, which was therefore more representative of breast cancer demographics. Hierarchical clustering over the common regions of alteration identified a novel subtype of high-grade estrogen receptor (ER)-negative breast cancer, characterized by a low genomic instability index. We were able to validate the existence of this genomic subtype in one external breast cancer cohort. Using matched array expression data we also identified the genomic regions showing the strongest coordinate expression changes ('hotspots'). We show that several of these hotspots are located in the phosphatome, kinome and chromatinome, and harbor members of the 122-breast cancer CAN-list. Furthermore, we identify frequently amplified hotspots on 8q22.3 (*EDD1*, *WDSOF1*), 8q24.11-13 (*THRAP6*, *DCC1*, *SQLE*, *SPG8*) and 11q14.1 (*NDUFC2*, *ALG8*, *USP35*) associated with significantly worse prognosis. Amplification of any of these regions identified 37 samples with significantly worse overall survival (hazard ratio (HR) = 2.3 (1.3-1.4) *p *= 0.003) and time to distant metastasis (HR = 2.6 (1.4-5.1) *p *= 0.004) independently of NPI.

**Conclusion:**

We present strong evidence for the existence of a novel subtype of high-grade ER-negative tumors that is characterized by a low genomic instability index. We also provide a genome-wide list of common copy number alteration regions in breast cancer that show strong coordinate aberrant expression, and further identify novel frequently amplified regions that correlate with poor prognosis. Many of the genes associated with these regions represent likely novel oncogenes or tumor suppressors.

## Abstract 2

High resolution array-CGH and expression profiling identifies a novel genomic subtype of ER negative breast cancer, and provides a genome-wide list of common copy number alterations associated with aberrant expression and poor prognosis.

## Background

High-resolution genome-wide profiling is allowing the copy number alterations underlying a wide range of distinct tumor types to be studied with unprecedented detail. Arguably, the most important insight to be gained from these studies is the identification of genomic regions harboring candidate oncogenes or tumor suppressors. A standard informatic approach has been to determine the regions of common gain (amplification) and loss (deletion) and then to correlate the copy number pattern of these regions with the mRNA expression patterns of genes contained in these loci. The association between gene dosage and expression levels is important and, as already shown in several studies, a significant proportion of gene expression variation can be explained in terms of underlying copy number alterations [1-3]. A further important insight gained through array comparative genomic hybridization (aCGH) data has been the identification of clinically relevant tumor subclasses within specific tumor types (e.g. myelomas [3], glioblastomas [4], pancreatic adenocarcinomas [5], colorectal cancer [2], etc.), which often match those found from genome-wide gene expression studies.

In breast cancer, most aCGH studies have used bacterial artificial chromosome (BAC) arrays [6-11] of at most 1 Mb resolution, cDNA arrays [1,12] or representational oligo arrays [13]. So far, the largest study combining copy number and gene expression data profiled 145 primary breast tumors derived from a heavily treated California patient population (henceforth called 'CAL') and which focused on tumors of relatively large size and high Nottingham Prognostic Index (NPI) [6] (see Table 1). This study supported the molecular taxonomy observed previously [1,10,12] and also identified many potential novel therapeutic targets. However, we asked whether the molecular taxonomy as well as the clinically relevant amplification and deregulation patterns could differ substantially if a tumor panel that is more representative of breast cancer demographics had been used. To this end, we performed a high-resolution (<100 kb) CGH study using a validated genome-wide oligo-based array [14] to profile a total of 171 primary breast tumors (the 'NCH' cohort) drawn from a tumor panel with NPI and tumor size distributions that were significantly different from previous cohorts (Table 1). In addition, we profiled 49 breast cancer cell lines. The aims of our work were twofold: first, to explore the taxonomy of breast tumors as defined at the copy number level and, second, to provide a comprehensive list of candidate oncogenes and tumor suppressors in breast cancer. To help us identify these genes we made use of a large accompanying gene expression data set profiling 113 of these tumors [15].

**Table 1:** Summary clinical table

	NCH (*n *= 171)	CAL (*n *= 145)	*p*	Sorlie (*n *= 85)	*p*	Porter (*n *= 44)	*p*
ER+	113 (66%)	96 (66%)		56 (76%)		29 (66%)	
ER-	57 (34%)	49 (34%)	1	18 (24%)	0.176	15 (34%)	1

Grade							
I	41 (24%)	16 (11%)		9 (12%)		12 (27%)	
II	57 (34%)	56 (40%)		33 (44%)		23 (52%)	
III	72 (42%)	69 (49%)	0.014	33 (44%)	0.063	9 (20%)	0.016

LN+	51 (30%)	74 (51%)		53 (70%)		11 (28%)	
LN-	120 (70%)	71 (49%)	0.0001	23 (30%)	<10^-8^	25 (62%)	1

Age	58 (57.1)	53 (55.4)	0.075	57 (57.8)	0.692	61 (59.5)	0.07

Size (cm)	1.8 (1.9)	2.2 (2.4)	0.0003	NA		2 (2.4)	0.67
≤ 1	12 (7%)	8 (6%)		NA		9 (22%)	
> 1, ≤ 2	109 (64%)	64 (45%)		NA		14 (34%)	
> 2, ≤ 5	49 (29%)	65 (46%)		NA		13 (32%)	
> 5	0 (0%)	5 (3%)	0.003	NA		5 (11%)	< 10^-6^

NPI	4.3 (3.9)	4.5 (4.7)	< 10^-7^	NA		NA	
< 3	34 (20%)	8 (6%)		NA		NA	
> 3, < 4	43 (25%)	22 (16%)		NA		NA	
> 4, < 5	65 (38%)	50 (36%)		NA		NA	
> 5	28 (16%)	58 (42%)	< 10^-6^	NA		NA	

Therapy							
None	79 (47%)	16 (11%)		0 (0%)		NA	
HT or CT	89 (53%)	128 (89%)	< 10^-11^	85 (100%)	< 10^-16^	NA	

A comparison is provided between the most important clinical parameters of the breast cancer cohort analysed in this study ('NCH') and three additional breast cancer cohorts 'CAL' [6], 'Sorlie' [12] and 'Porter' [11]. For estrogen receptor status (ER), Grade, lymph node status (LN) and Therapy received (HT = hormone therapy, CT = chemotherapy), *p *values were computed using Fisher's exact test. For age, tumor size and the NPI (Nottingham Prognostic Index) we give the median (and mean) values and the *p *values obtained using a Wilcoxon rank sum test. For tumor size and NPI we also give the distributions across various thresholds and the corresponding *χ*^2 ^test *p *values.

## Results

### Preprocessing

Details concerning the aCGH profiling of the samples and subsequent normalization can be found in Materials and Methods. Detailed clinical data of the breast cancer cohort profiled is available in Additional Data File 1, while the raw and normalized aCGH data for tumors and cell lines is available from NCBI's Gene Expression Omnibus (GEO) [16-18] under the series accession number GSE8757. Briefly, after segmentation of the mode-normalized data using the CBS algorithm [19], we applied the method described in [5] to define thresholds for gain and loss. We observed that because the cellularity of samples varied widely (mean cellularity, expressed as percentage, was 69% with a standard deviation of 19%), the genome instability index (GII; defined as the fraction of genome altered) was highly correlated with cellularity (Additional Data File 2, panel A). To correct for this unwanted effect without sacrificing a considerable number of samples, thresholds were redefined separately for each sample using a cellularity correction model similar to the model described in [20] (see also Materials and Methods). After correction, the GII became independent of cellularity (Additional Data File 2, panel B), thus validating the approach we adopted. The choice of thresholds was further validated with the help of breast tumor cell lines with known gains and losses. Thresholds for amplification were initially defined for cell-lines with known amplicons and rescaled for primary tumors using the cellularity correction (see Materials and Methods).

To test our normalization and segmentation further, we evaluated the concordance of alteration patterns between the oligo array and a genosensor BAC array, on which 126 of the 171 breast tumors had been previously profiled [10] (see also Materials and Methods). After matching the locations of the oligos to the 281 BACs representing cancer-related loci, we found a strong concordance between both types of copy number data (28 of the 34 matched regions, 82%, showed strong agreement with a Fisher-exact test *p *< 0.05; see Additional Data File 2, panel C). A similar degree of good concordance between BAC and oligo data was recently observed across a panel of 19 prostate cancers [21].

### Genomewide patterns of gain and loss

**Figure 1:**
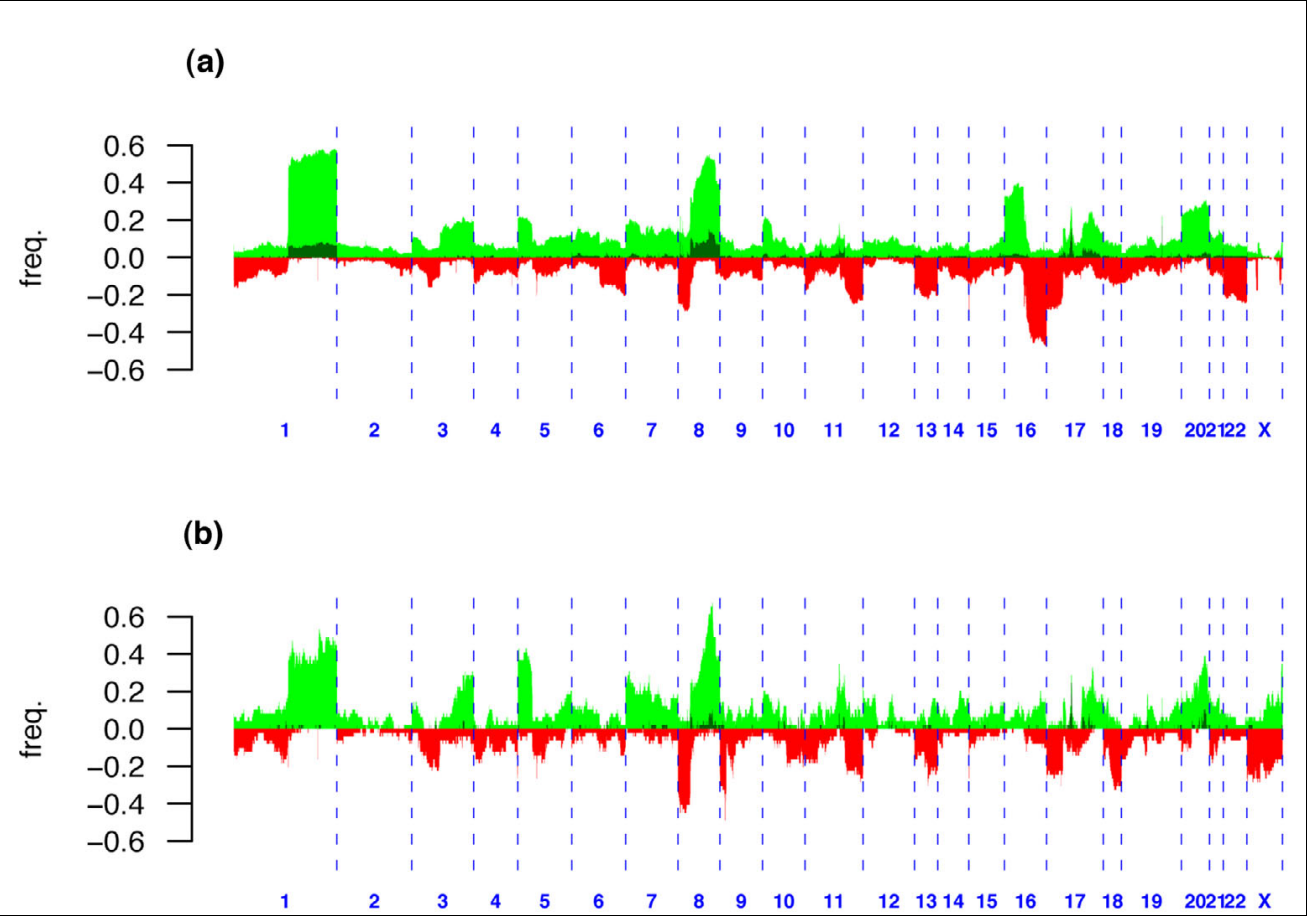
**Genome-wide frequency plots**. Genome-wide frequency plot of gains (green), amplifications (darkgreen) and loss (red) over: **(a)**, 171 primary breast tumors; and **(b)**, 49 breast cancer cell lines.

Genomewide patterns of gain and loss showed a significant number of highly recurrent altered regions (Figure 1 and Additional Data File 3). The patterns for tumors and cell lines were remarkably similar to each other and in concordance with previously published studies [1,6,7,13]. Interestingly, the pattern was also similar to that reported for lung cancer [22]. In brief, chromosomal regions that were most commonly gained in both tumors and cell lines were 1q21.1-qtel, 5ptel-5p13.3, 8p12-8q24.3, 17q12, 17q21-17q25.1 and 20q11-qtel. Chromosomal regions that were most commonly lost in both tumors and cell lines were 8ptel-8p12, 11q14-qtel, 13q21-qtel and 17ptel-17p11.2. However, there were also notable differences between tumors and cell lines. Specifically, cell lines showed a higher frequency of losses on chromosomes 9, 18 and X, and a lower frequency of losses on 16q, as compared with tumors. On the other hand, tumors showed a higher frequency of gains on 16p. In agreement with [6] we observed regions of recurrent high-level amplification on chromosomes 8, 11, 12, 17 and 20 (Figure 1a) bounding well-known breast cancer oncogenes (e.g. *BRF2*, *ASH2L*, *CCND1*, *EMSY*, *ERBB2*, *NCOA3*, *MYBL2*, *STK6*) [10,23,24], although amplification frequencies were much lower on chromosomes 12 and 20 as compared with those reported in [6]. In contrast, cell lines did show amplification frequencies on chromosomes 12 and 20 that were more in line with those observed in [6] (Figure 1b). We found homozygous deletion (HD) to be a rare event in primary tumors and only found evidence of HD in two cell lines and one tumor on chromosome 13q14 where the retinoblastoma gene (RB-1) resides.

### Common and minimal regions of alteration

To perform dimensional reduction we developed an extension (CRalg) of the minimal regions algorithm of Rouveirol (MRalg) [25], which, in contrast to MRalg, identifies common regions of alteration (CRA) (see Materials and Methods). Using CRalg we achieved a substantial dimensional reduction (from 27695 oligos to 5914 CRA that showed at least 5% changes across tumors) without losing any information in the process (note that the MRalg and CRalg algorithms will work unchanged if instead of using 1 and -1 to indicate gain and loss, we used the precise segment values; thus, CRalg achieves a dimensional reduction without further information loss), automatically including gains and losses in the same matrix. However, a drawback of CRalg was the relatively larger number of variables (5914 CRA compared with 1134 minimal regions of alteration (MRA)) and the high degree of redundancy/correlation since many adjacent CRA only differed in value in one sample. In order to reduce the redundancy of the CRA matrix, we applied an algorithm that merged together adjacent regions that differed in only a single sample (see Materials and Methods). This gave a reduced matrix of 1063 merged CRA (mCRA) over 171 breast tumors.

### A subgroup of low GII

**Figure 2:**
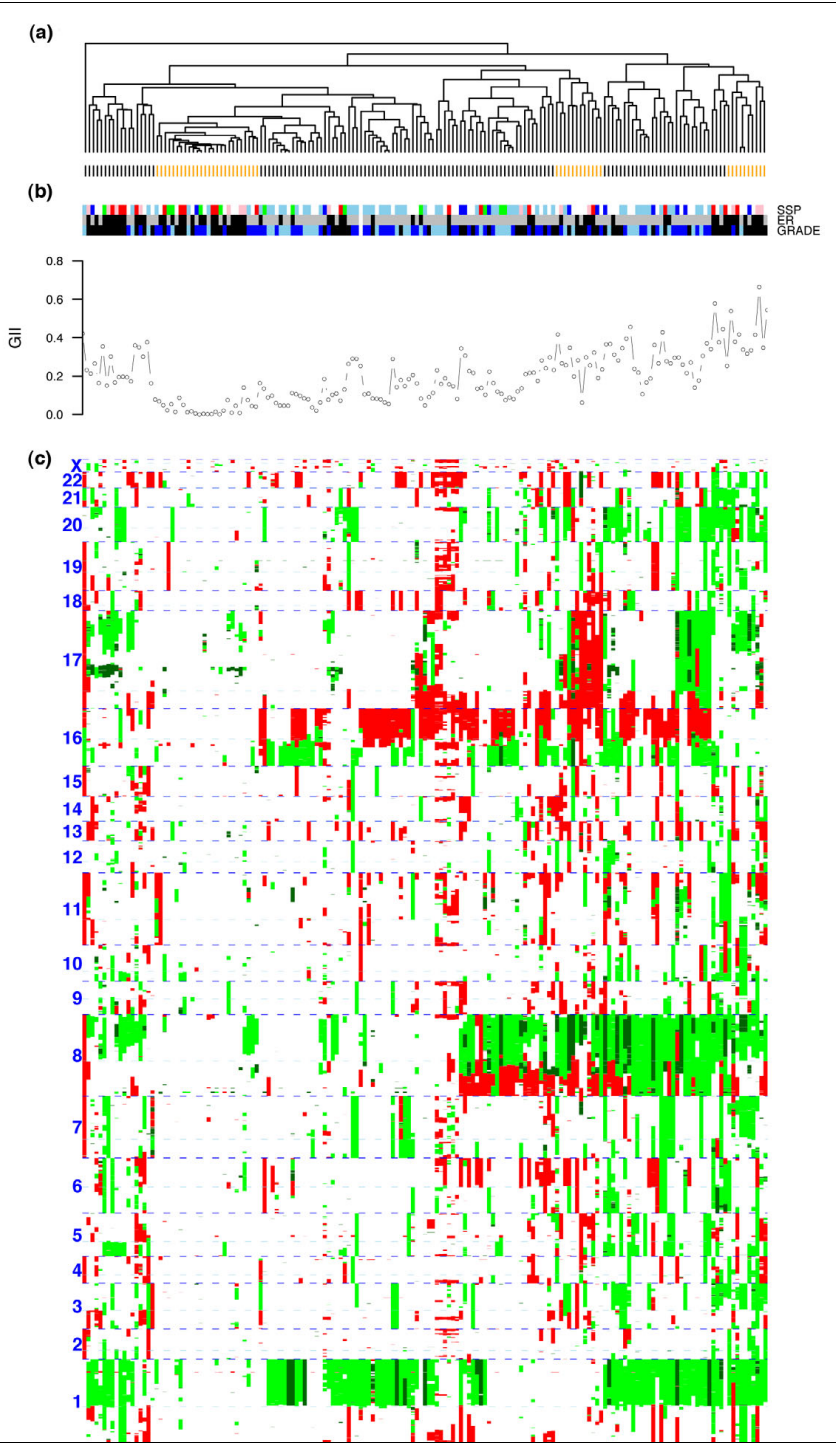
**Unsupervised clustering of 171 breast tumors**. **(a)**, Hierarchical clustering over 1063 merged CRA using complete linkage and number of copy-number state differences as a distance metric. Clusters labeled in orange denote the largest stable clusters as determined by the *pvclust *algorithm. **(b)**, Associated sample distributions of intrinsic subtype based on the SSP classifier (sky blue, luminal-A; blue, luminal-B; green, normal; red, basal; pink, HER2), ER status (black, ER-; gray, ER+), grade (black, grade III; blue, grade II; sky blue, grade I) and GII. **(c)**, Heatmap of CRA (dark green, amplification; green, gain; white, normal; red, loss).

While standard hierarchical clustering algorithms have been successfully applied to BAC-derived continuous log-ratio data, we explored the possibility of incorporating the inherent discreteness of copy number data into the unsupervised classification analysis. Specifically, we performed (complete linkage) hierarchical clustering over the matrix of mCRA using the number of copy number state differences as a distance metric. This revealed a complex pattern of gains and loss across the cohort (Figure 2). Using the methodology implemented in the *R*-package *pvclust *[26,27] for testing the robustness of the clusters, we found that only one reasonably sized cluster of 26 samples was reliable with a robustness index larger than 90% (Figure 2 and Additional Data File 4). This cluster was characterized by a very low GII (average of 0.036 ± 0.035) relative to the rest of samples (average of 0.22 ± 0.12), which was highly significant (Wilcoxon test *p *< 10^-13^). We verified that this result was independent of cellularity by showing that this cluster did not have a significantly lower cellularity than the rest of samples (Wilcoxon rank sum test *p *= 0.69). The 26-sample cluster was made up of proportionally more ER-negative (15) than ER-positive tumors (11) (Fisher-exact test *p *= 0.007) as well as more basal (6) than luminal tumors (5) (Fisher-exact test *p *= 0.01), but was equally distributed in terms of histological grade (3 grade I, 10 grade II and 13 grade III, *p *= 0.28), the immunohistochemical markers ERBB2, PGR, AR and p53, and p53 mutation status (21 samples with no p53 mutation and 4 with p53 mutation, *p *= 0.79). Among the 26 samples there were 8 with gains of *ERBB2 *and 5 of these had a high-level *ERBB2 *amplification. This confirms the observation made in [10] that a proportion of *ERBB2*-amplifier tumors show little overall genomic instability.

Two further, yet much smaller, clusters with robustness indices greater than 90% and of relatively high GII were also identified (Figure 2). The cluster with the highest GII was made up of 9 samples and was mainly characterized by gains of 1q, 8q, telomeric end of 17q and 20, and unaltered chromosome 16. Most of the samples were ER negative (6 ER- versus 3 ER+) and of high grade (7 grade III, 1 grade II and 1 grade I). Another robust cluster of 12 samples and intermediate GII was characterized mainly by loss of chromosome 17, loss of 16q and gain of 8p. This cluster was made up of 7 ER+ and 5 ER- samples and was also mostly high grade (7 grade III, 3 grade II and 2 grade I). The rest of samples could not be characterized as members of large stable clusters.

### A novel subtype of ER- tumors of low genomic instability

**Figure 3:**
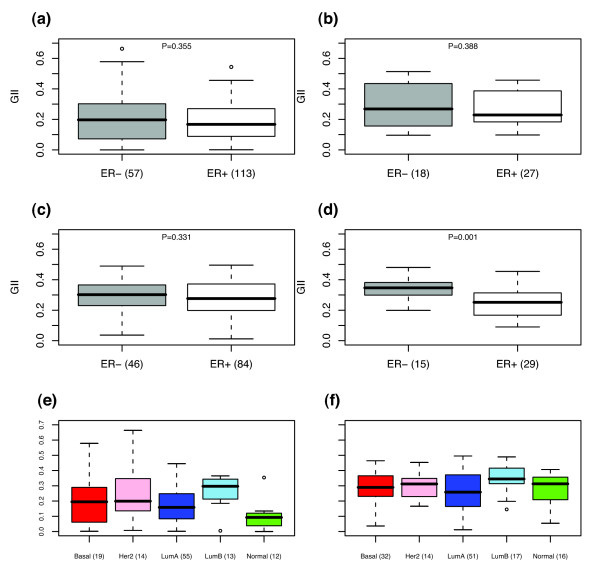
**Distributions of genomic instability index**. Boxplots of the distribution of GII across different ER and expression subtypes: **(a)**, **(e)**, NCH cohort; **(b)**, Naylor's cohort [9]; **(c)**, **(f)**, CAL cohort [6]; **(d)**, Loo's cohort [11].

The identification of a subclass of breast tumors of low genomic instability that was proportionally enriched in terms of ER- and basal tumors was striking and suggested to us that, in contrast to present belief, there is a subtype of ER- tumors of relatively low genomic instability and which includes a subset of *ERBB2*-amplifier tumors. Further evidence for this came from a Wilcoxon rank sum test comparing the GII distributions of ER- and ER+ samples, which showed that the GII of ER- samples was not significantly higher than that for ER+ samples (Figure 3a, *p *= 0.35). Importantly, among the 15 ER-samples within the 26 sample low-GII subgroup, 10 were of high grade, 4 of intermediate grade and only 1 of low grade, which was proportionally similar to the distribution in the rest of the ER- cohort (30 high grade, 9 intermediate grade and 3 low grade tumors, *p *= 0.88). This showed that the ER- samples in the low GII cluster were not necessarily of lower grade.

To obtain further evidence for the existence of a low GII ER- subtype, we sought independent validation in three external breast cancer cohorts [6,9,11] for which copy number data was available. Specifically, we computed the GII in these external cohorts as described in Materials and Methods and tested, using a one-sided Wilcoxon rank sum test, whether there was a substantial number of ER- samples of relatively low GII (Figure 3b, c and 3d). Lending further support to the existence of this low-GII subtype, in two of these external cohorts [6,9] we did not find the GII of ER- samples to be significantly higher than that for ER+ samples.

In terms of the intrinsic subtype classification [28-30], for which a single sample predictor (SSP) was recently derived and validated in external cohorts [31], we found that the 26-sample low-GII subgroup was made up of 6 basal, 3 HER2+, 4 luminal-A, 4 normal and 1 luminal-B tumors (8 samples could not be classified owing to missing gene expression information). As before, when taking into account all samples, the basal subtype did not have a significantly higher GII than the luminal-A subtype (*p *= 0.44) (Figure 3e). We interpreted this result as further evidence for the existence of a low-GII basal subtype. The only statistically significant differences between the GII distributions of the various intrinsic subtypes were between the normal subtype and all others (*p *< 0.05 for all comparisons) and between the luminal-A and luminal-B subtypes (*p *= 0.009). We observed a similar GII distribution in another cohort for which expression data was available [6] (Figure 3f). Specifically, in this cohort as well, the basal subtype did not have a significantly higher GII than the luminal-A subtype (*p *= 0.26), while the luminal-B subtype did (*p *= 0.03).

### The low-GII subgroup has an associated gene expression signature

To further characterize the identified low-GII subgroup, we attempted to derive an associated transcriptomic signature from the 113 samples for which additional gene expression information was available. To this end we used a multiple logistic regression model and ranked genes according to the difference of their model Akaike information criterion (AIC) score [32] with respect to a null model AIC score that only included ER status (see Materials and Methods). The null distribution for AIC scores was obtained by performing 10000 random permutations of the sample expression values. Hence, this method allowed us to rank the genes according to how well they discriminated between the 26-sample low-GII cluster and the rest of the cohort, independently of ER status. To correct for multiple testing we converted the *p *values into *q*-values [33], which provided us with an estimate of the false discovery rate (FDR). This showed that, for example, among the top-50 genes we would expect on average about 10 false positives, thus confirming the existence of an expression signature associated with this subclass.

To derive a classifier based on this gene signature we decided on a linear discriminant classifier where class assignment is determined by a nearest centroid criterion using an euclidean distance metric. The centroids were constructed using the top-37 genes (Additional Data File 5), yielding an average of 7 false positives. To test this classifier we first applied it to the 135 NCH samples with gene expression information [15]. This classified 15 ER- and 9 ER+ into the putatively low-GII subgroup (25 ER- and 84 ER+ were classified into the other group), which we verified had a lower GII than the rest of the samples (Wilcoxon test *p *< 10^-4^). It is striking that even though the classifier was derived independently of ER status, classifying for this particular subgroup of low GII predetermined samples to be more likely ER- than ER+ (Fisher-exact test *p *= 0.0003). Interestingly, applying the classifier to four additional breast cancer cohorts with expression profiles [6,34-36] showed that the corresponding putatively low-GII subgroup in these cohorts was also enriched for ER- tumors (Additional Data File 6). In a further data set [30] where only 85 samples (18 ER- and 56 ER+) were available the predicted low-GII subclass had only 4 ER+ and 3 ER- samples (and 3 samples had missing ER information), which did not reach statistical significance, but suggested to us that it possibly would if more samples were available.

**Figure 4:**
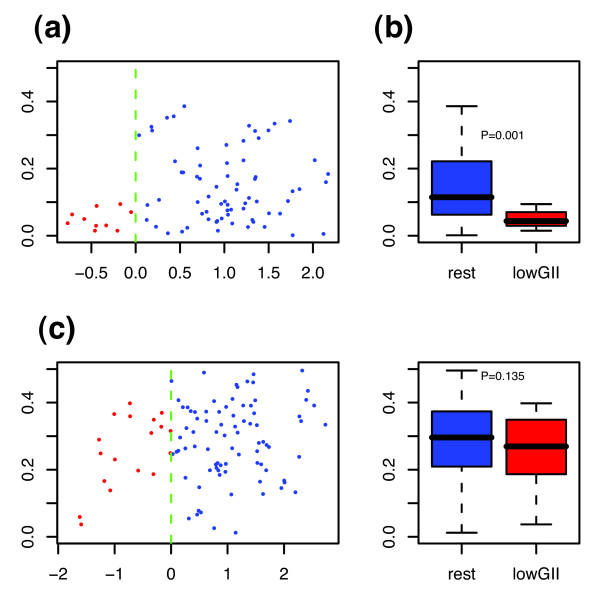
**Genomic instability index versus LD-scores**. **(a)**, **(c)**, GII is plotted against the linear discriminant (LD) scores for the 86 samples profiled in [12] and the 101 samples of the CAL cohort [6]. Those samples with a negative LD score were classified into the low-GII subgroup (red), the rest are shown in blue. **(b)**, **(d)**, Corresponding boxplots showing the GII distributions of the two predicted subgroups.

Fortunately, the tumors in [30] were profiled recently at the copy number level [12]. This allowed us to validate the hypothesis that our gene expression classifier selects a particular subtype of low GII in breast cancer. Using the GII for the samples in this cohort we compared the GII of the predicted low-GII subgroup, as determined by our expression classifier, with the rest of samples in that cohort (Figure 4a and 4B). In spite of only 10 samples being classified into the predicted low-GII subgroup, we could verify that it was characterized by a lower GII when compared with the rest of the samples (Wilcoxon test *p *= 0.001). Moreover, among these 10 samples, 6 were of high grade, 2 of intermediate grade and none were of low grade (2 samples had missing information). For the other external cohort for which both copy number and expression data was available [6], the predicted low-GII subgroup had a lower median GII than the rest of samples, but did not reach statistical significance (Figure 4c and 4D).

To better understand the nature of the expression classifier we performed both gene ontology (GO) analysis using GOTM [37] and pathway analysis using MSigDB [38]. GOTM on the 37 genes making up the classifier showed enrichment of inflammatory and defense response genes (*CXCL1*, *CXCL2*, *XCR1*, *LY96*, *NMI*, *TLR2*, uncorrected *p *< 10^-5^), which were generally upregulated in the low-GII subgroup, and marginal enrichment of signal transduction (*RASSF2*, *SNX4*, *CASP1*, *MKNK1*, *RHPN1*, *INPP5D*, uncorrected *p *= 0.002) and apopotosis genes (*BCL2A1*, *MRPS30*, *CASP1*, *CASP4*, *TLR2*, uncorrected *p *= 0.004). Pathway analysis using MSigDB confirmed the involvement of the caspase, cell death, TNF-α-NF-κβ, inflammatory response and signalling pathways, although these statistical associations were lost on correction for multiple testing (data not shown).

### Gene expression and copy number

Of the 171 breast tumors, 113 were also profiled on Agilent gene expression arrays [15]. This allowed us to evaluate the contribution of gene-dosage levels to gene expression (Additional Data File 7). Of the 5914 CRA, 4551 (77%) contained at least one Agilent probe. Of these 4551 CRA, 2407 harbored at least one Agilent probe for which there was at least 10 (~5%) expression values in the altered (i.e. gained or lost) group of samples (note that owing to missing values in the gene expression data, *p *values could not be reliably computed for many probes). Thus, for 2407 CRA at least one reliable *p *value (Wilcoxon test) could be computed (see Materials and Methods) to evaluate the significance of the association between copy number and aberrant expression. We found that from the 2407 CRA, there were 806 CRA for which there was at least one probe with significant association (*p *< 0.05) between gain and overexpression, and 412 for which there was at least one probe with significant association between loss and underexpression. On average about 34% of probes in regions that were gained in at least 5% of samples were significantly overexpressed relative to the samples that showed no copy number alteration. Similarly, about 29% of probes in regions that were lost in at least 5% of the samples were significantly underexpressed relative to the samples that showed no copy number alteration. This confirms the finding reported elsewhere [1] that a significant proportion of gene expression variation is caused by underlying copy number alterations.

### Hotspots of association between copy number and expression

To find the CRA showing the strongest associations between copy number and expression we first tabulated those CRA with at least 10% gains or losses and which showed a significant association with expression (*p *< 0.05; see Additional Data File 8). To narrow this down to a smaller set of the most significant regions ('hotspots') we next selected those CRA with an association index (AI) value larger than or equal to 0.5 and a most significant *p *value of less than 0.001, where the AI was defined as the fraction of probes within the CRA that had significant *p *values (see Methods). This yielded 196 and 63 hotspots that showed significant association with overexpression and underexpression, respectively (Additional Data File 9). In the case of loss and associated underexpression this table included the well-known tumor suppressors *RB1*, *CDH1*, *MBD2 *and *EP300*, while in the case of gain and overexpression it included many well-known and potentially novel oncogenes such as *MUC1 *on 1q21.3, *ASH2L*, *BRF2*, *LSM1 *on 8p12, *FADD *on 11q13, *ERBB2*, *PNMT*, *GRB7 *on 17q12, *TOP2A*, *THRA*, *NR1D1 *on 17q21, and *NCOA6*, *YWHAB*, *UBE2C *on 20q13. Of these candidate oncogenes, several, notably *TOP2A*, *PNMT *and *UBE2C*, have appeared in prognostic gene expression signatures [39-41], thus re-emphasizing their important role in breast cancer. Among the hotspots that were gained, we provide a further selection of those that also showed frequent amplifications and which are therefore likely to harbor candidate oncogenes (Table 2).

**Table 2:** Hotspots of gain and amplification

CytoBand	Start	Length	Gains (T)	nAMP (T)	Gains (CL)	nAMP (CL)	Genes
1q21.1	144.22	1.65	0.49	10	0.37	0	*RBM8A, POLR3C, ZNF364*
1q21.3-1q22	153.29	0.09	0.51	8	0.39	0	*EFNA4, MUC1*
1q23.2	157.95	0.32	0.53	10	0.39	1	*DUSP23, IGSF9*
1q23.3	159.23	<0.01	0.55	12	0.43	1	*F11R*
1q42.13-1q42.2	226.95	2.59	0.57	12	0.45	0	*SPHAR, NUP133, GALNT2*
1q43-1q44	240.32	4.75	0.58	11	0.45	0	*SDCCAG8, ADSS, FAM36A*
6q21	107.12	<0.01	0.11	5	0.02	0	*AIM1*
6q21	107.14	0.2	0.11	4	0.02	0	*RTN4IP1, QRSL1*
8p12	37.82	<0.01	0.32	16	0.24	4	*GPR124, BRF2*
8p12	38.04	0.1	0.32	17	0.22	3	*ASH2L, LSM1*
8p12	38.24	0.06	0.32	18	0.24	3	*WHSC1L1*
8q21.13	82.73	0.14	0.47	13	0.37	1	*ZFAND1, CHMP4C*
8q21.3	87.54	0.48	0.49	15	0.39	1	*FAM82B, CPNE3*
8q22.3	102.57	1.37	0.54	17	0.51	1	GRHL2, RRM2B, EDD1*, AZIN1*
8q22.3	104.52	<0.01	0.54	15	0.51	1	WDSOF1*
8q24.11	118.02	0.58	0.54	23	0.61	4	THRAP6*
8q24.12	120.81	0.1	0.53	23	0.57	3	DCC1*
8q24.13	124.90	1.14	0.53	22	0.65	3	TRMT12*, RNF139*, NDUFB9*
8q24.13	126.06	0.28	0.53	23	0.65	4	SQLE*, KIAA0196*
8q24.3	144.53	0.09	0.40	12	0.39	0	*RHPN1, ZC3H3*
9p22.3-9p22.1	16.40	2.65	0.11	1	0.12	1	*C9orf39, FAM29A*
10p14	12.20	0.05	0.20	2	0.18	0	*NUDT5, SEC61A2*
10p13	12.33	2.66	0.20	3	0.18	0	*OPTN, FAM107B, SUV39H2*
11q13.3	69.73	<0.01	0.17	11	0.33	2	*FADD*
11q13.3	69.90	<0.01	0.16	8	0.31	1	*PPFIA1*
11q14.1	77.01	0.38	0.12	8	0.24	3	*CLNS1A, INT4*
11q14.1	77.47	0.16	0.12	10	0.29	3	NDUFC2*, ALG8*, USP35*
16p13.2-16p13.13	8.78	1.68	0.38	3	0.06	0	*ABAT, PMM2, USP7*
16p12.3	18.44	1.21	0.39	3	0.10	0	*NOMO2, ARL6IP, MIR16*
17q12	33.73	<0.01	0.16	9	0.08	3	*MRPL45*
17q12	34.15	0.02	0.19	15	0.12	4	*PSMB3*
17q12	34.19	0.02	0.19	15	0.12	4	*CCDC49*
17q12	34.33	0.01	0.20	16	0.12	4	*LASP1*
17q12	34.67	0.01	0.23	23	0.12	4	*FBXL20*
17q12	35.06	0.02	0.27	25	0.29	11	*STARD3, TCAP, PNMT*
17q12	35.08	0.06	0.27	27	0.29	12	*ERBB2, GRB7*
17q12	35.29	0.03	0.23	21	0.27	10	*GSDML*
17q21.1	35.44	0.07	0.16	15	0.16	5	*THRAP4, NR1D1*
17q21.2	35.77	0.03	0.13	6	0.06	1	*TOP2A*
17q21.32	44.27	0.1	0.17	9	0.22	3	*ATP5G1, UBE2Z*
17q21.33	44.95	0.19	0.18	6	0.18	2	*SPOP, SLC35B1*
17q21.33	45.80	0.02	0.19	7	0.20	2	*MRPL27, LRRC59*
17q22	54.12	0.44	0.19	6	0.20	2	*RAD51C, TRIM37*
17q23.1	55.12	0.01	0.23	9	0.20	2	*CLTC, PTRH2*
17q23.1	55.38	0.1	0.23	8	0.20	3	*RPS6KB1, ABC1*
17q23.3	58.97	0.29	0.23	6	0.20	1	*CCDC44, DDX42, FTSJ3*
17q24.2	62.77	<0.01	0.24	7	0.33	1	*PSMD12*
17q25.1	68.72	0.03	0.22	4	0.18	0	*COG1, C17orf80*
17q25.1	70.52	0.03	0.17	5	0.16	0	*ICT1, ATP5H*
17q25.1	70.57	0.2	0.16	5	0.16	0	*HN1, NUP85, MRPS7*
17q25.1	71.15	0.15	0.17	4	0.16	0	*ITGB4, H3F3B*
20p11.23	17.97	1.51	0.25	3	0.16	0	*ZNF133, RBBP9, HARS2*
20q13.12	42.95	0.05	0.27	2	0.29	2	*YWHAB*
20q13.12	43.77	0.2	0.26	1	0.29	0	*DNTTIP1, UBE2C, NEURL2*
20q13.13	46.43	0.86	0.28	2	0.29	1	*CSE1L, STAU1*
20q13.33	60.21	0.53	0.28	3	0.31	0	*HRH3, ADRM1, LAMA5*
20q13.33	61.45	0.2	0.27	3	0.18	0	*EEF1A2, C20orf149, PTK6*
20q13.33	62.04	0.17	0.27	3	0.20	1	*UCKL1, C20orf14, OPRL1*
21q22.3	42.85	0.47	0.13	2	0.08	0	*SLC37A1, WDR4, NDUFV3*
21q22.3	46.49	0.2	0.12	2	0.06	0	*MCM3AP*

Selected regions of frequent gain/amplification and strong coordinate overexpression. Columns give cytoband, start position (Mb), length (Mb), frequency of gains across 171 tumors (T), number of amplifications in 171 tumors (T), frequency of gains across 49 cell lines (CL), number of amplifications in 49 cell lines, selected genes in region showing strong coordinate overexpression. * denotes genes significantly associated with either overall survival, time to distant metastasis or disease free interval (*p *< 0.05).

### Hotspots associated with outcome

As the identified 196 and 63 hotspots represent the regions of strongest association between copy number and coordinate aberrant expression, it was natural to investigate whether any of these regions also showed association with clinical outcome. To this end we performed univariate Cox proportional hazard regressions comparing the HRs for samples with gain (loss) of a hotspot with samples without altered hotspots for three different outcome endpoints (overall survival (OS), disease free interval (DFI) and time to distant metastasis (TTDM); see Additional Data File 9). In addition, we performed Cox regressions for those hotspots with at least 10 amplifications and estimated the HR for samples with and without amplification (Additional Data File 9). This analysis showed that there were three cytoband regions of frequent amplification (8q22.3, 8q24.11-8q24.13 and 11q14) and associated with either OS or TTDM (log-rank test *p *< 0.05; see Table 3). We verified that for all of these regions samples with amplification had approximately a twofold risk increase of poor outcome compared with samples without the amplification (Table 3). Interestingly, 37 tumors had amplifications in any one of these hotspot regions and this subgroup had also a much higher risk of distant metastasis and a poorer survival rate compared with samples without amplification (TTDM HR = 3.0 (1.6-5.8) *p *< 10^-3^; see Table 3). Genes located in these hotspots, which also showed coordinate overexpression, included *EDD1, WDSOF1 *(8q22.3), *THRAP6 *(8q24.11), *DCC1 *(8q24.12), *SQLE*, *KIAA0196 *(8q24.13) and *NDUFC2*, *ALG8*, *USP35 *(11q14.1) (Tables 2 and 3 and Additional Data File 9). Importantly, multivariate Cox regression analysis in a model that included the NPI (one of the strongest prognostic factors in univariate analysis), showed that amplification of any of these five regions was a strong prognostic factor independently of NPI (Table 4).

**Table 3:** Amplification hotspots associated with clinical outcome

Cytoband	Genes	nAMP	OS HR (95% CI) *p *value	TTDM HR (95% CI) *p *value
8q22.3	*EDD1, AZIN1*	17	2.2 (1.1-4.6) 0.02	2.1 (0.9-5) 0.09
8q22.3	*WDSOF1*	15	2.2 (1.1-4.7) 0.03	1.9 (0.7-4.9) 0.17
8q24.11	*THRAP6*	23	1.9 (1-3.8) 0.04	2.1 (0.9-4.5) 0.06
8q24.12	*DCC1, DEPDC6*	23	1.9 (1-3.7) 0.05	2.1 (0.9-4.5) 0.06
8q24.13	*SQLE, SPG8*	23	2 (1-3.9) 0.03	2.2 (1-4.7) 0.05
11q14.1	*NDUFC2, ALG8, USP35*	10	2.5 (1.1-6) 0.03	2.4 (0.8-6.8) 0.09
5-amp	5-amp	37	2.6 (1.5-4.6) 3 × 10^-4^	3.0 (1.6-5.8) 5 × 10^-4^

Amplification hotspots significantly associated with either overall survival (OS) or time to distant metastasis (TTDM). The cytoband locations, genes located in amplified hotspots that showed coordinate overexpression, the number of samples with amplification (nAMP), hazard ratio (HR), 95% confidence interval and log-rank test *p *values are given.

**Table 4:** Univariate and multivariate survival analysis

Factor	OS HR (95% CI) *p *value	TTDM HR (95% CI) *p *value
ER	1.7 (0.9-2.5) 0.10	1.7 (0.8-3.3) 0.13
p53mut	1.7 (0.9-3.0) 0.09	2.1 (1.1-4.2) 0.03
LN	2.4 (1.4-4.0) 8 × 10^-4^	4.3 (2.3-8.3) < 10^-4^
Size	1.3 (1.0-1.7) 0.03	1.3 (0.9-1.7) 0.16
Grade	2.0 (1.2-3.4) 0.009	1.8 (0.9-3.3) 0.08
NPI	2.5 (1.4-4.3) 0.001	2.8 (1.4-5.8) 0.003
5-amp	2.6 (1.5-4.6) 3 × 10^-4^	3.0 (1.6-5.8) 5 × 10^-4^

5-amp*+NPI	2.3 (1.3-4) 0.003	2.6 (1.4-5.1) 0.004
5-amp+NPI*	2.2 (1.2-3.9) 0.008	2.5 (1.2-5.1) 0.02

Univariate and multivariate Cox proportional hazards analysis with overall survival (OS) and time to distant metastasis (TTDM) as endpoints. Univariate analysis was performed for ER status (1, negative; 0, positive), p53 mutation (1, mutant; 0, wild-type), lymph node status (1, positive; 0, negative), size (1, ≥2.5 cm; 0, <2.5 cm), grade (1, high or intermediate; 0, low), NPI (1, ≥3.8; 0, <3.8) and 5-amp (1, amplification in any of the five regions; 0, no amplification in any region). * indicates the corresponding hazard ratio estimate in the multivariate model that included 5-amp and NPI.

### Gene families

Next, we investigated the patterns of gain and loss for particular gene families, including the kinome [42], the phosphatome [43], a selected set of chromatin binding proteins and chromatin modifier enzymes that we collectively called 'chromatinome', and the list of somatically mutated breast cancer genes (CAN genes) [44]. The relevance of these gene families and gene sets for cancer biology is well known [42,44,45]. Specifically, we investigated whether there was preferential selection for genomic changes among these gene sets (see Materials and Methods), and also which genes showed significant coordinate aberrant expression.

#### CAN genes

Of the 122 genes that were shown to be somatically mutated at a higher frequency in breast cancer [44], 121 were found on the oligo CGH array. As expected, many of the CAN genes (e.g. *TP53*, *TMPRSS6 *and *APC2*) were frequently lost, but many also showed frequent gains (e.g. *PTPN14*, *NCOA6 *and *HOXA3*; see Additional Data File 10). Analysis of preferential selection for genomic changes showed, not unexpectedly, that CAN genes were more frequently lost in comparison with random selections of 121 genes with the same chromosome distribution (*p *< 0.05), while there was no preferential selection for gains (*p *= 0.84). Of the 121 genes on the aCGH array, 108 were also mapped on the Agilent array and 9 showed significant association between expression and copy number, including *NCOA6, OBSCN *and *DDX10 *(Additional Data File 11).

#### Phosphatome

Of the 107 phosphatases described in [43], 90 were mapped onto the oligo CGH and Agilent arrays and 10 showed significant association between copy number and expression (Additional Data File 11). Among the class I Cys-based protein tyrosine phosphatases (PTPs), the subclass of 16 myotubularins were frequently lost in comparison with the rest of phosphatases, with *MTMR2 *also showing coordinate underexpression relative to samples with no loss. The analysis for preferential selection for genomic changes showed that phosphatases were more frequently lost (*p *< 0.05), while gains were not selected (*p *= 0.96).

#### Chromatinome

We compiled a list of 503 histones, chromatin binding proteins and chromatin modifier enzymes, of which 440 were also found mapped on the Agilent array. These genes did not show preferential selection for either gains (*p *= 0.67) or losses (*p *= 0.97). Of these 440, 51 showed significant association between copy number and expression (Additional Data File 11). For example, we found that *HDAC2 *and *ASH2L *showed coordinated aberrant expression in samples for which the gene was either gained or lost, while samples with gains of *CREBBP *and *SUV39H2 *showed significant overexpression compared with samples that showed no corresponding copy number alteration. In addition, *EP300 *was found to be lost in over 20% of tumors with a corresponding significantly lower expression compared with tumors for which the gene copy number was not altered. These observations are particularly noteworthy given that chromatin modifiers are infrequently mutated in breast cancer [46-48].

#### Kinome

Of the 518 kinases reported in [42], 477 were found to be in CRA and 268 of these were also mapped on the Agilent array. Out of these 268 kinases, 32 exhibited significant association between copy number and gene expression (Additional Data File 11). Notably, *ERBB2 *showed the strongest association between copy number gain and overexpression, followed by kinases on chromosome 1, *CLK2 *and *SCYL2*, and *RIPK2 *on chromosome 8. As far as loss and underexpression is concerned, the strongest associations were found for *MAP2K4*, *NEK3*, *TESK1 *and *MLKL *on chromosomes 17, 13, 9 and 16, respectively. Of note, we observed that the association of *MAP2K4 *loss with underexpression is consistent with observations that it may play a role as a tumor suppressor [49]. While, individually, kinases were frequently altered and showed coordinate gene expression changes, we did not observe any differences in the frequency of alterations between the nine kinase families *AGC, CAMK, CK1, CMGC, RGC, STE, TK, TKL *and *Atypical*, nor preferential selection for gains (*p *= 0.96) or losses (*p *= 0.17).

## Discussion

Many mRNA profiling studies have established that breast cancer is a highly heterogeneous disease with at least five identified 'intrinsic' subtypes [30,50], and recent evidence points at the likely existence of additional biologically and/or clinically relevant subtypes [31,51]. As changes in copy number drive a considerable proportion of the changes at the transcriptomic level [1], it is likely that the aberration landscape underlying breast cancer at the copy number level is of an even far more complex nature than that observed at the mRNA level. Exacerbating this complexity further is the fact that a significant proportion of genomic aberrations are totally unrelated to cancer physiology and merely reflect random events that differ between any two normal specimens [52].

The aCGH study presented here is the largest study to date to combine copy number and expression data, having profiled 171 primary breast tumors with a high-density oligo array, and while it confirms the findings reported recently in [6,10,13], it also shows that breast cancer is a more heterogeneous disease than is portrayed by these previous studies. Specifically, we found, using hierarchical clustering with a novel distance measure, only three robust clusters of 10 or more samples, the largest of which, with 26 samples, was characterized by a low GII and was surprisingly enriched for ER- and basal samples. The other two clusters also consisted mainly of intermediate/high grade ER- tumors, but were characterized by a high GII. These findings suggested to us the existence of a high-grade ER-/basal subgroup of low GII. In agreement with this conclusion, we observed in two additional independent cohorts that ER-tumors, despite being of higher grade than ER+ tumours, did not have a higher GII. An analogous result was also obtained when considering the basal and luminal status of the tumors. Moreover, while in ER+/luminal tumors a subdivision into high and low GII can be explained by the differential distribution of histological grade (larger GII for high-grade tumors) [6], no such grade association seems to explain the variability/bimodality in GII that is observed for ER- tumors. It is also noteworthy that while the subdivision into high and low GII that is observed for ER+ tumors correlates with clinical outcome and with the luminal-A and luminal-B subtypes, no such correlation with clinical outcome is observed in the case of ER- tumors.

More generally, we investigated the distribution of other clinical phenotypes (age, tumor size, vascular invasion, NPI, lymph node status, distant metastasis, overall survival, p53 mutation status and the immunohistochemical markers PGR, ERBB2, p53 and AR) in the 26-sample low-GII cluster relative to the rest of the cohort, as well as the differential distribution of the same clinical factors among the two groups when restricted to ER- samples only. No strong associations were found, although the low-GII subgroup was proportionally enriched for lymph node negative (LN-) patients: 22 LN- and four 4 LN+ in the low-GII subgroup relative to 98 LN- and 47 LN+, Fisher *p *= 0.10, when considering all samples; and 13 LN- and 2 LN+ in the low-GII subgroup relative to 25 LN- and 18 LN+, Fisher *p *= 0.06, when restricted to ER- samples only.

Thus, in order to better characterize the identified low-GII subgroup of 26 samples we derived an expression classifier using a subset of 113 samples for which expression data was available. The expression classifier was derived independently of ER status and was successfully validated in one of the two external cohorts for which both expression and copy number data was available [12]. Moreover, using additional independent expression data sets we were able to show that the expression classifier selects mostly ER-tumors. When combined, these results provide strong evidence that the derived transcriptomic signature is a classifier of low-GII ER- samples. We can only speculate as to why the expression classifier did not select for low-GII samples in the other external cohort for which both copy number and expression data was available [6], although in agreement with the other studies it did select for ER- samples (Additional Data File 6). One possible explanation could be the much higher GII values of the cohort in [6] compared with those in our cohort (Figure 3). Interestingly, we also found that the median tumor size was considerably larger in [6] compared with our cohort (2.2 cm compared with 1.8 cm), which was highly significant under a Wilcoxon rank sum test (one-sided test *p *< 10^-5^). Thus, by selecting a panel of relatively large ER+ and ER- tumors, the study in [6] may have missed out on this ER- subtype of low GII. Similarly, the tumors profiled in [11] were significantly larger than those in our cohort (Table 1) and, correspondingly, we also observed relatively higher GII values in their cohort (Figure 3). To investigate this possibility further we asked whether there was a significant correlation between tumor size and genomic instability (Additional Data File 12). Strikingly, in our cohort as well as the cohorts in [6] (CAL) and [11] (Porter) we observed a step-like structure in the distribution of GII and tumor sizes (Additional Data File 12). Without exception, we observed that there were no tumors of sizes larger than 2.5 cm and GII values lower than 0.1. Using 2.5 cm as a cut-off, we verified that the GII of tumors of larger size (i.e. ≥2.5 cm) were significantly higher than those of smaller tumors (<2.5 cm) (Wilcoxon rank sum test, *p *= 0.017 (NCH), *p *= 0.018 (CAL), *p *= 0.18 (Porter)). Together these findings indicate that our identification of a low-GII subgroup was facilitated by the smaller sizes of the NCH cohort in comparison with the cohorts profiled elsewhere. A similar observation could also be made in relation to the study in [13], which profiled significantly larger tumors and estimated only 10% of tumors to have 'flat' (i.e. low-GII) profiles, in comparison with the 30% of tumors with a GII of less than 0.1 in the NCH cohort. (This must be interpreted with caution as the authors in [13] did not define their 'flat' profiles in terms of GII values.)

Gene ontology analysis of the 37-gene expression classifier showed marginal statistical associations with inflammatory response, apoptosis and signal transduction genes. Similarly, pathway analysis showed that the most enriched pathways were those related to caspase activity, cell death, NFκB, immune function and signal transduction. Interestingly, *BCL2A1*, a known transcriptional target of NFκB, was found to be upregulated in the low-GII subgroup, which is consistent with the observed upregulation of the inflammatory response genes (e.g. *CXCL1*, *CXCL2*, *LY96*) which may mediate the NFκB activation.

The combined copy number expression analysis further confirmed the presence of many genomic regions with expression aberrations that are driven by underlying copy number changes [1,6]. Of the nine candidate therapeutic targets reported to be frequently amplified and deregulated at the expression level [6], we were able to verify six of these (*IKBKB, ERBB2, ADAM9, FNTA, PNMT *and *NR1D1*) (Additional Data File 8). Of these, *ERBB2*, *FNTA*, *PNMT* and *NR1D1* were located in hotspots that showed particulary strong associations between copy number gain and overexpression (Additional Data File 9). Interestingly, however, hotspots that were frequently amplified and that were associated with clinical outcome did not include the regions 8p11-12 and 17q11-12 reported in [6]. Instead, we found that hotspots associated with either survival or time to distant metastasis were located on cytobands 8q22.3, 8q24.3, 8q24.11-13 and 11q14, involving other important breast cancer genes such as *EDD1, WDSOF1 *(8q22.3), *THRAP6 *(8q24.11), *DCC1 *(8q24.12), *SQLE*, *KIAA0196 *(8q24.13) and *NDUFC2, ALG8, USP35 *(11q14.1) (Table 2 and Additional Data File 9). Specifically, *SQLE *expression has been shown to be a robust prognostic marker [39,50], and *WDSOF1 *was part of the gene expression predictor derived in [53]. The genes on cytoband 11q14.1, *NDUFC2, ALG8 *and *USP35*, also reside close to what appears to be a novel amplicon in acute myeloid leukemias (AML) [54]. The different clinically relevant hotspot regions identified here in comparison with those found in [6] may be a consequence of the different clinical characteristics of the two cohorts, but more likely it reflects the substantial differences in treatment (Table 1). Specifically, in the 'NCH' cohort only 53% of tumors received either hormone or chemotherapy (and only six, i.e. 4%, received chemotherapy) in comparison to the 'CAL' cohort where almost 90% of patients received treatment (Table 1). Thus, the combined analysis of copy number, expression and clinical outcome variables in a patient population with almost 50% untreated cases and better overall prognostic variables, has identified potentially novel clinically relevant amplicons in breast cancer.

## Conclusion

By profiling a large panel of relatively small and low-NPI breast tumors that is representative of breast cancer demographics in the UK we have shown that high-grade ER-/basal breast cancer can be subdivided into two subclasses of low and high genomic instability. In addition, we provide a comprehensive list of hotspot genomic regions that show strong correlation between copy number and expression, and have identified novel candidate amplicons associated with poor prognosis independently of standard prognostic factors, including the NPI.

## Materials and methods

### Primary tumor genomic DNA and cell lines

Primary breast tumor specimens were obtained with appropriate ethical approval from the Nottingham Tenovus Primary Breast Cancer Series. All 171 cases were primary operable invasive breast carcinomas collected from 1990 to 1996. Whole tissue sections (tumor cellularity range 20-100%) were used for DNA extraction. Detailed clinical data for this cohort is available (Additional Data File 1). The 49 breast cancer cell lines were obtained from the American Type Tissue Collection (Manassas, VA) or were generously provided by their originator. The cell lines were cultured according to the culture conditions recommended by their providers. The normal reference pools were established using peripheral blood from 10 anonymous donors (with ethical approval).

### DNA isolation and labelling

DNA was extracted from 20 sections of 30 μm from each tumor using the Promega DNA Wizard kit (Promega, UK) according to manufacturer's instructions. DNA was extracted from cell lines and peripheral blood leukocytes using standard SDS/Proteinase K method. DNA was quantified with a NanoDrop ND-1000 spectrophotometer (NanoDrop Technologies, Wilmington, DE, USA). DNA labelling was performed using the BioPrime DNA labelling kit reagents (Invitrogen) and according to protocols described previously [14].

### aCGH data preprocessing and normalization

Labelled DNAs were hybridized to customized oligonucleotide microarrays containing 30000 60-mer oligo probes [14], for which 27801 unique map positions were defined (Human Mar. 2006 assembly (hg18)). The median interval between mapped elements was 39.4 kb, 75% of intervals were less than 104.2 kb and 95% were less than 402 kb. Fluorescence ratios of scanned images of arrays were obtained using BlueFuse version 3.2 (Bluegnome). Raw aCGH profiles of 171 breast tumors and 49 cell lines were then processed using the *R*/Bioconductor package *limma *[55]. Mode normalizations were subsequently carried out for all arrays. The raw and mode-normalized data for the 171 tumors and 49 breast cell lines is available from NCBI's GEO [16-18] under the series accession number GSE8757.

### Identification of copy number transitions

The normalized aCGH data was then segmented using the CBS algorithm [19] as implemented in the *R*-package *DNAcopy *[19]. The CBS algorithm parameters used were: number of permutations 5000, window size 500 and overlap 0.5. Next, we fitted a density to the distribution of segmented state values and verified that the resulting mode was close to zero. The segmented data was then recentered by shifting the position of this mode to zero. This yielded a matrix of segment values for 27801 unique probes and 171 tumor samples.

### Thresholds for calling gains, losses, amplifications and deletions

Having identified the segments and the baseline of unaltered copy number, we next applied an extension of the algorithm in [5] for calling gains and losses. As the cellularity of the tumor samples varied significantly across the cohort, we extended Aguirre's method to take the cellularity of the samples into account. Thus, sample-specific thresholds were obtained. Specifically, the procedure used was as follows.

1. The mode-normalized log-ratios were first transformed back to ratios. The ratio values for sample *s*, *R*_*gs*_, were then corrected for sample cellularity *c*_*s*_, by the transformation

$${\tilde{R}}_{gs}=\frac{1}{c_{s}}(R_{gs}-(1-c_{s}))$$

2. Next, we log_2 _transformed back the corrected ratio values and computed the standard deviation, *σ*_*s*_, of the middle 50% quantile of the data.

3. A threshold for calling gain and loss for sample s was then defined as ${\tilde{t}}_{s}=\pm 2\sigma_{s}$.

Note that for the estimation of the thresholds only the middle 50% quantile of the data is needed, thus in step 1 above negative ${\tilde{R}}_{gs}$ values were generally avoided. Subsequently, the transformation in step 1 was applied to all of the inverse log_2 _transformed segment values providing a further regularization of the data.

In a few cases where negative values were obtained, given the monotonicity of the transformations, these states were treated as a loss of copy number.

For amplifications we used a threshold of 8*σ*_*s *_(i.e. four times the threshold for one-copy gains). With this choice of thresholds we verified that *ERBB2 *had frequencies of gain and amplification of 0.27 and 0.15, respectively, which are close to the frequency values quoted in previous studies [1,10].

In the case of cell lines, thresholds for gain and loss were defined at ± 0.25 on a log_2 _scale and were close to the average threshold values over cell lines obtained by the above procedure using *c*_*s *_= 1 (specifically, the average was 0.20 was gains and -0.27 for losses). As before, the amplification threshold was defined as four times the threshold for gain (i.e. at 1 on a log_2 _scale).

### Concordance between oligo and BAC arrays

Of the 171 breast tumors, 126 had been previously profiled on a Genosensor (Vysis, Downer's Grove, USA) BAC array [10] for DNA copy number aberrations. This BAC array contained 281 unique BAC clones representing cancer-related loci. When we matched the locations of the 27801 unique clones in the high-resolution oligo-array to the 281 BAC clones in the Genosensor array, 34 BACs were found to contain at least 5 oligos. Concordance between oligo and BAC arrays was evaluated by examining their discrete copy number states in the matching regions. DNA copy number status (gain(1), loss(-1), normal (0)) for both oligo and BAC arrays were assigned for the above 34 matching regions/clones. A Fisher-exact test was then used to determine the association between the two types of arrays for each of the 34 matching regions.

### CRA and MRA

The matrix of segmented values is not useful for many of the downstream analyses, such as candidate oncogene identification and unsupervised classification. Hence, from the matrix of segmented values, we derived different data matrices with different downstream applications in mind.

For the purpose of identifying a list of candidate oncogenes and tumor suppressors, we applied the algorithm of [25] to define the minimal regions of gain and loss (MRG and MRL). This algorithm requires as input matrices of gains and losses over all of the probes, which we constructed using the thresholds for gain and loss as described above. As explained in [25], the minimal regions define the shortest regions that are commonly gained or lost across the cohort. While this algorithm captures those regions most likely to harbor candidate oncogenes and tumor suppressors, the algorithm may also fail to capture known oncogenes or tumor suppressors. This can happen due to several reasons. One reason is the sensitivity of the algorithm to errors in the segmentation algorithm or perturbations in the sample set. Alternatively, it might also fail to capture more complex patterns of amplification or deletion involving multiple neighboring targets. Thus, in addition to deriving the minimal regions we also applied a different algorithm (CRalg) which considers all breakpoints equally. Similar to the algorithm of [25] (MRalg), it captures the regions that are commonly gained or lost across the cohort, but in contrast to MRalg, it not only captures the minimal regions but also all other, usually adjacent, regions of gain and loss. Specifically, following the notation of [25], we have the following theorem that applies to CRalg.

**Theorem 1 ***A region r *= [*in *... *out*] *is a CRA if and only if*

(i) *'in' and 'out' are breakpoints; and*

(ii) *there is no breakpoint b such that in *<*b *<*out*.

Thus, CRalg encapsulates all of the information from the matrix of segmented values into a much smaller number of variables, while MRalg loses potentially important information. Clearly, many adjacent CRA will be highly correlated, only differing in value across one of the samples. In order to remove this redundancy we applied a merging step to the CRA. Thus, adjacent regions that only differed in value in one sample were merged together. For every sample, we defined the value for the newly merged region as the median value over all of the regions merged together. Thus, if three regions are being merged with values (1, 1, 0) for that sample, the newly merged region would have value 1. If only two regions are merged with values say (1, 0) then a value of 0.5 would be assigned for the newly merged region. Thus, this approach allowed us to reduce the number of correlated variables significantly, while also retaining as much information as possible.

### GII

We defined the GII of a sample as the fraction of its genome that was altered. This index was computed in two different ways, which showed very strong concordance (Spearman rank correlation 0.96). In one method we computed it as the fraction of the genome that was altered based on the CRA. In the second method we estimated it as the fraction of altered oligos, where the corresponding segment value was used to determine the altered status of each oligo.

For the three external data sets, we used the GII as provided by [9], while for the other two [6,12] data sets the GII was computed from the normalized segmented data using a method similar to that which we used (but without correcting for cellularity, since this was unavailable).

### Transcriptomic characterization of the low-GII subgroup

To characterize the identified subgroup of low GII, which was enriched for ER- tumors, at the transcriptomic level we used the following procedure. For each of the genes *g *for which expression data was available, we fitted a multiple logistic regression model of the form *TYPE *~ *EXP*(*g*) + *ER*, where *TYPE *denotes the type of sample according to the bi-partite clustering (low-GII subgroup = 2; rest = 1), *EXP*(*g*) denotes the expression vector of the gene *g *and *ER *denotes the ER status of the samples. To evaluate how well a gene could discriminate between samples according to the clustering type over and above the prediction by ER status alone, we compared the AIC scores of the multiple logistic regression model in relation to a null AIC score distribution obtained by 10,000 random permutations of the sample expression values. Specifically, genes were ranked in order of increasing AIC (low AIC means better model fit) and a *p *value of significance was estimated by counting the number of null AIC values lower than the observed value. The computations were performed using the neural network *R*-package *nnet *[27]. The *p *values were then converted into *q *values using the *q-value R*-package [33].

### Gene expression and copy number

To evaluate genome-wide correlations between gene expression (profiled on Agilent) and copy number we followed an approach similar to that in [56] and which is based on the Wilcoxon test. Briefly, probes for which gene expression measurements were available were tested for associations between dosage and expression levels by comparing the distribution of expression values for altered (i.e. either gained or lost) versus unaltered samples. The criterion used to decide whether a *p *value could be computed for a given probe was based on setting a threshold on the minimum number of gene expression values present. Specifically, we counted for each probe the number of available expression values among samples that had the corresponding region altered (note that owing to missing values in the gene expression matrix, the number of available expression values was not the same as the number of altered samples). If there were at least 10 samples (~5%) where the genomic region was gained and for which corresponding gene expression values were available we computed a *p *value using the Wilcoxon test to evaluate significance of association between copy number gain and overexpression. Similarly, we used a 10 sample (~5%) threshold for evaluating significance between copy number loss and underexpression.

To identify the hotspots of strongest association between expression and copy number we first computed the AI for each CRA, defined as the fraction of probes within the CRA with Wilcoxon test *p *values less than 0.05. We then filtered CRA on a per-chromosome basis by selecting those with AI ≥ 0.5 and having a most significant *p *value less than 10^-3^. Setting a threshold on the most significant *p *value was necessary to remove a large number of CRA containing only one significant expression measurement (for which AI = 1).

### Gene families

To evaluate whether there was preferential selection for genomic changes in the gene families (CAN genes, kinome, phosphatome and chromatinome), we compared the frequencies of gain and loss of a given gene family (*n *members) with that of a randomly selected set of *n *genes with the same distribution across chromosomes as the original family set. While this is a conservative procedure it nevertheless enabled us to evaluate the significance of the alteration frequencies relative to the expected frequencies within each chromosome. A total of 1000 Monte Carlo randomizations were used and the comparison of the alteration frequency distributions was done in two alternative ways, (i) by comparing the means of these distributions and (ii) with one-sided Wilcoxon rank-sum tests, both of which were found to be entirely consistent. By computing the fraction of Monte Carlo runs where the mean from the randomized distribution was larger than the observed value, a *p *value could be estimated.

## Additional data files

The following Additional data files are available with the online version of this paper: Additional data file 1 is a text file showing the clinical table for the 171 breast tumors of the NCH cohort; Additional data file 2 is a PDF file showing GII against cellularity for the 171 breast tumors; Additional data file 3 is an Excel file listing the common regions of most frequent gain and loss (10% gain/loss threshold) for tumors and cell lines separately. Additional data file 4 is a PDF file depicting the cluster stability analysis of the hierarchical clustering over the 1063 merged regions and 171 breast tumor samples, using the R-package pvclust. Additional data file 5 is a PDF file showing the centroid of expression for the low-GII subgroup identified in Figure 2. Additional data file 6 is a  PDF file of the expression classifier for the low-GII subgroup in four independent external breast cancer cohorts. Additional data file 7 is a PDF file showing a Chromosome by Chromosome plot of (i) the frequency of gain (green) and loss (red) profiles of CRA over the 171 tumors, and (ii) the p values (log10 scale) of Agilent probes in these regions that evaluate the association between copy number gain and overexpression (green), or loss and underexpression (red).  Additional data file 8 is a subset of tables of ADF-3 (tumors only) listing the CRA that showed significant association between copy number gain and overexpression or copy number loss and underexpression whereas Additional data file 9 shows tables listing the CRA that showed strong statistical association ('hotspots') between either copy number gain and overexpression or copy number loss and underexpression within the previous subset.  Additional data file 10 is a PDF file depicting Frequency of gains (green) and loss (red) for the most frequently altered CAN genes.  Additional data file 11 is a PDF file showing tables of CAN genes, 'kinome' genes, 'phosphatome' genes and 'chromatinome' genes frequently altered across breast tumors and also showing coordinate aberrant expression.  Finally, Additional data file 12 is a PDF file showing GII plotted against tumor size for the Nottingham City Hospital cohort profiled in this study (NCH) (red), the cohort from California (CAL) profiled in [6] (black) and the cohort (Porter) profiled in [11] (pink).

## Authors' contributions

SFC performed the profiling experiment. The statistical analysis was carried out by AET, JCM and YW with input from NPT, MAVDW and ST. The platform gene annotation was performed by NLBM. JLC assisted with the CGH profiling experiments. The principal tumor set was obtained from AGR and IOE. IHC scoring was performed by AGR and SEP. PLP contributed an independent breast tumor cohort profiled at the copy number level. The study was conceived by CC, BY and JDB. AET and CC wrote the manuscript with input from SFC, JCM and YW.

## Supplementary Material

Additional data file 1 Clinical table for the 171 breast tumors of the NCH cohort. Clinical factors are: ER, estrogen receptor status; GRADE, histological grade; NPI, Nottingham Prognostic Index; Size, tumor size (cm); VI, vascular invasion; Stage; Age; Cellularity, percentage of tumor cells; survival (time and event); DFI, disease free interval; RECC, recurrence(yes/no); TTLR, time to local recurrence; LR, local recurrence (yes/no); TTRR, time to regional recurrence; RR, regional recurrence (yes/no); TTDM, time to distant metastasis; DM, distant metastasis (yes/no); Endocrine, endocrine therapy received (yes/no); CT, chemotherapy received (yes/no). Click here for file

Additional data file 2 GII, defined as the fraction of genome altered, against cellularity for 171 breast tumors. A, GII before cellularity correction; B, GII after cellularity correction; C, for overlapping altered regions as determined by BAC and oligo arrays, we plot the *p *value of the one-sided Fisher-exact test evaluating the concordance of altered/unchanged states between BAC and oligo arrays (a low *p *value is representative of high concordance). Click here for file

Additional data file 3 Tables listing the common regions of most frequent gain and loss (10% gain/loss threshold) for tumors and cell lines separately. Columns label the index of the CRA, the genes (symbols) annotated to this region, the corresponding cytoband, start and end positions (Mb), length (Mb), number of mapped oligos in the region, frequency of gain, minimal region (1, yes; 0, no), number of oligos within this region and on the Agilent array with available expression data, fraction of these showing statistically significant coordinate aberrant expression, the corresponding best and worst *p *values, the genes showing significant correlation between copy number change and expression, and number of amplified cases. Click here for file

Additional data file 4 Cluster stability analysis of the hierarchical clustering over the 1063 merged regions and 171 breast tumor samples, using the *R*-package *pvclust*. The red numbers indicate the robustness index of the clusters. There was only one cluster with a robustness index larger than 90% and containing more than 20 samples. Click here for file

Additional data file 5 The centroid of expression for the low-GII subgroup identified in Figure 2. Genes were mean centered and standardized to unit variance. The top 37 genes discriminating between the low-GII subgroup and the rest of the samples are shown ranked from top to bottom together with their direction of differential expression. Click here for file

Additional data file 6 The expression classifier for the low-GII subgroup in four independent external breast cancer cohorts: A, van de Vijver *et al*. [35]; B, Wang *et al*. [34]; C, Sotiriou *et al*. [36]; and D, CAL [6]. Samples in the predicted putative low-GII subgroup are labeled in pink, the rest of the samples are shown in orange. The top color bar denotes ER status (black, ER-; gray, ER+). In the heatmaps, red denotes relative overexpression and green denotes relative underexpression. Click here for file

Additional data file 7 For each chromosome we plot (i) the frequency of gain (green) and loss (red) profiles of CRA over the 171 tumors, and (ii) the *p *values (log_10 _scale) of Agilent probes in these regions that evaluate the association between copy number gain and overexpression (green), or loss and underexpression (red). Threshold lines of 5% gain and 5% loss and 0.05 significance level are also shown (black). Click here for file

Additional data file 8 Subset tables of ADF-3 (tumors only) listing the CRA that showed significant association between copy number gain and overexpression or copy number loss and underexpression at a significance level *p *< 0.05. Click here for file

Additional data file 9 Subset tables of ADF-8 listing the CRA that showed strong statistical association ('hotspots') between either copy number gain and overexpression or copy number loss and underexpression. The criterion used for a region to be a hotspot was a best *p *value with *p *< 0.001 and at least 50% of oligos in the region showing an association between copy number state and aberrant expression at *p *< 0.05. Click here for file

Additional data file 10 Frequency of gains (green) and loss (red) for the most frequently altered CAN genes. Click here for file

Additional data file 11 Tables of CAN genes, 'kinome' genes, 'phosphatome' genes and 'chromatinome' genes frequently altered across breast tumors and also showing coordinate aberrant expression. Click here for file

Additional data file 12 GII is plotted against tumor size for the Nottingham City Hospital cohort profiled in this study (NCH) (red), the cohort from California (CAL) profiled in [6] (black) and the cohort (Porter) profiled in [11] (pink). Samples in the NCH cohort that clustered in the 26-sample low-GII subgroup are shown in green. Click here for file
