# Supplementary material for: High-resolution aCGH and expression profiling identifies a novel genomic subtype of ER negative breast cancer
Source: Genome Biol. 2007 Oct 7;8(10):R215. doi: 10.1186/gb-2007-8-10-r215 (PMC2246289; doi:10.1186/gb-2007-8-10-r215)
Supplement: Additional data file 2 — GII, defined as the fraction of genome altered, against cellularity for 171 breast tumors. A, GII before cellularity correction; B, GII after cellularity correction; C, for overlapping altered regions as determined by BAC and oligo arrays, we plot the p value of the one-sided Fisher-exact test evaluating the concordance of altered/unchanged states between BAC and oligo arrays (a low p value is representative of high concordance). [file gb-2007-8-10-r215-S2.pdf]

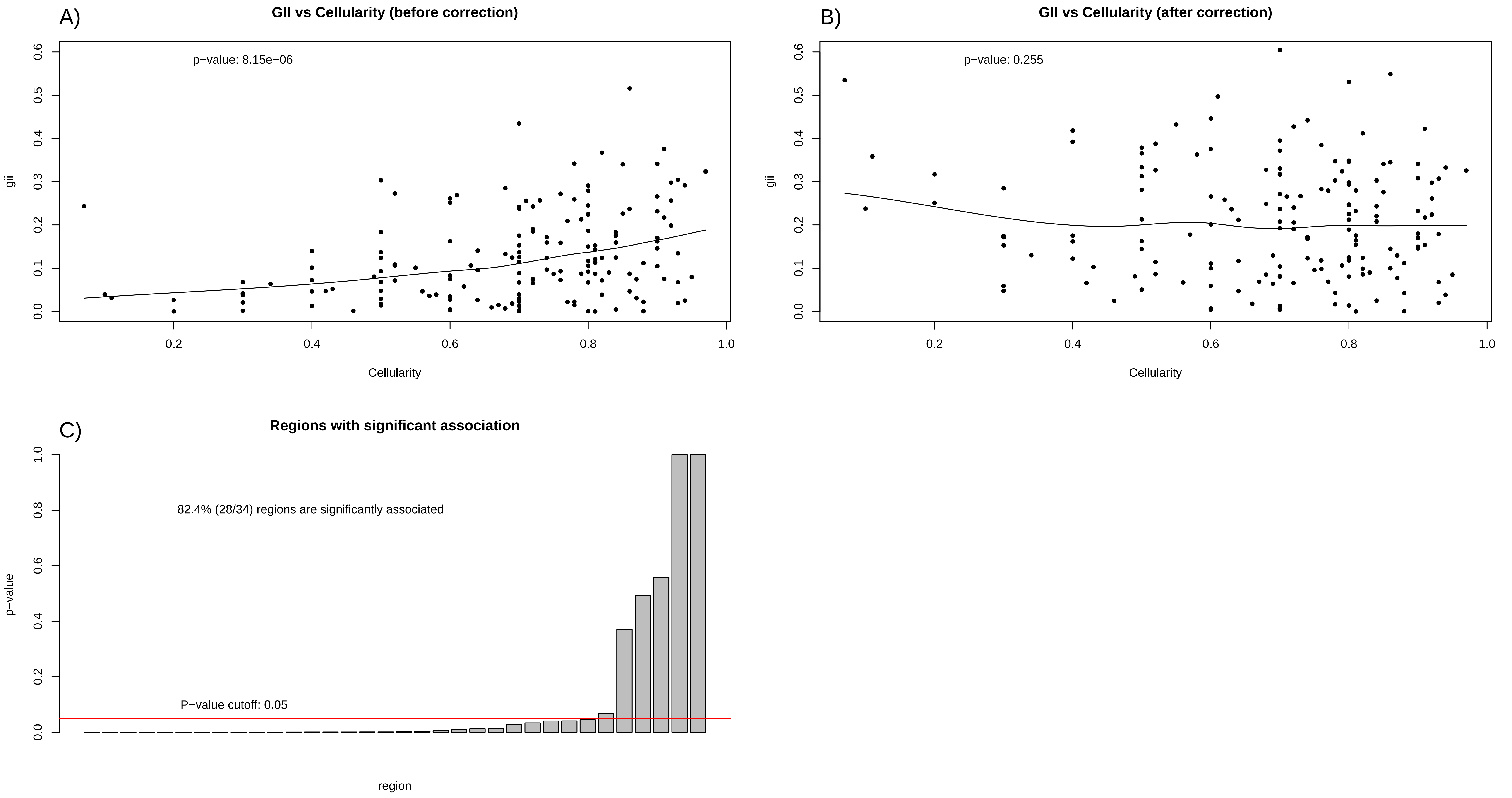
A)
GII vs Cellularity (before correction)
p−value: 8.15e−06
gii
Cellularity
B)
GII vs Cellularity (after correction)
p−value: 0.255
gii
Cellularity
C)
Regions with significant association
82.4% (28/34) regions are significantly associated
P−value cutoff: 0.05
p−value
region
