# Supplementary figures and images for: High-resolution aCGH and expression profiling identifies a novel genomic subtype of ER negative breast cancer
Source: Genome Biol. 2007 Oct 7;8(10):R215. doi: 10.1186/gb-2007-8-10-r215 (PMC2246289; doi:10.1186/gb-2007-8-10-r215)

## Low GII (18)

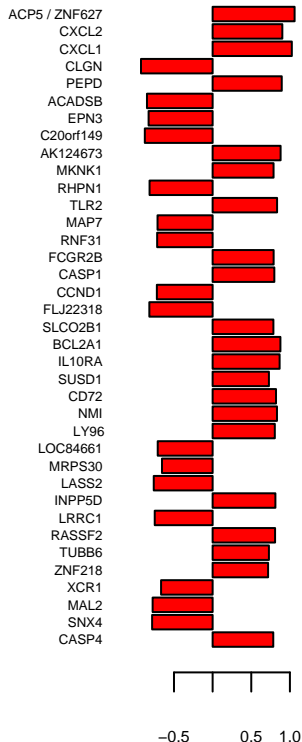

## Rest (95)

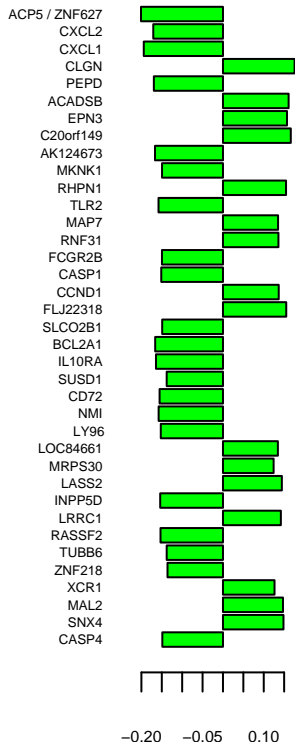

Supplement: Additional data file 5 — The centroid of expression for the low-GII subgroup identified in Figure 2. Genes were mean centered and standardized to unit variance. The top 37 genes discriminating between the low-GII subgroup and the rest of the samples are shown ranked from top to bottom together with their direction of differential expression. [file gb-2007-8-10-r215-S5.pdf]

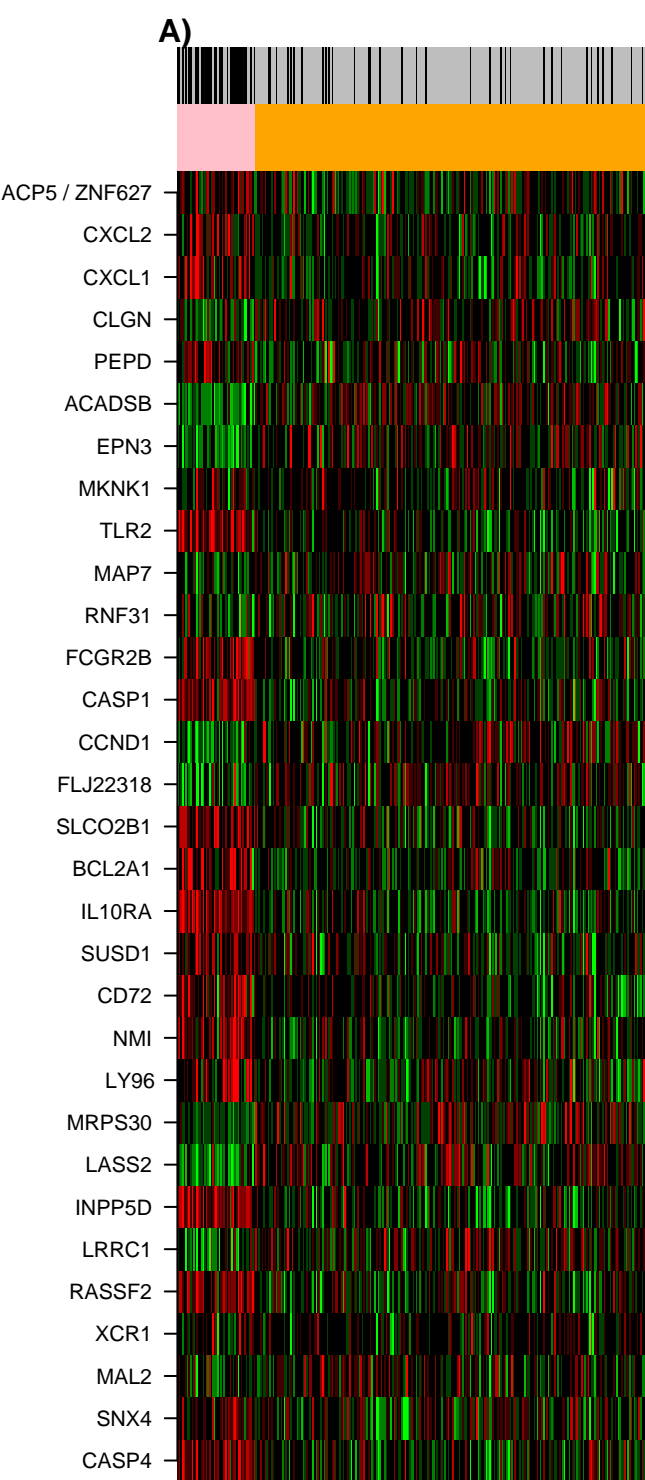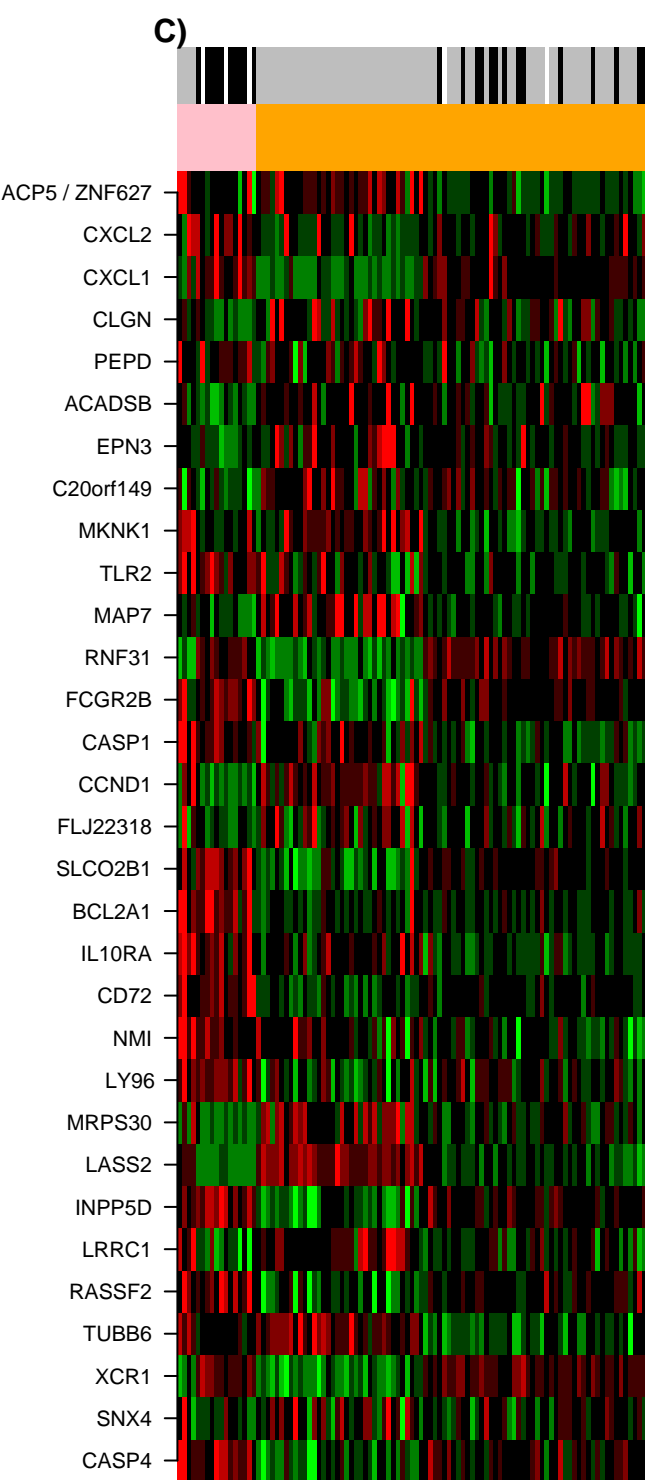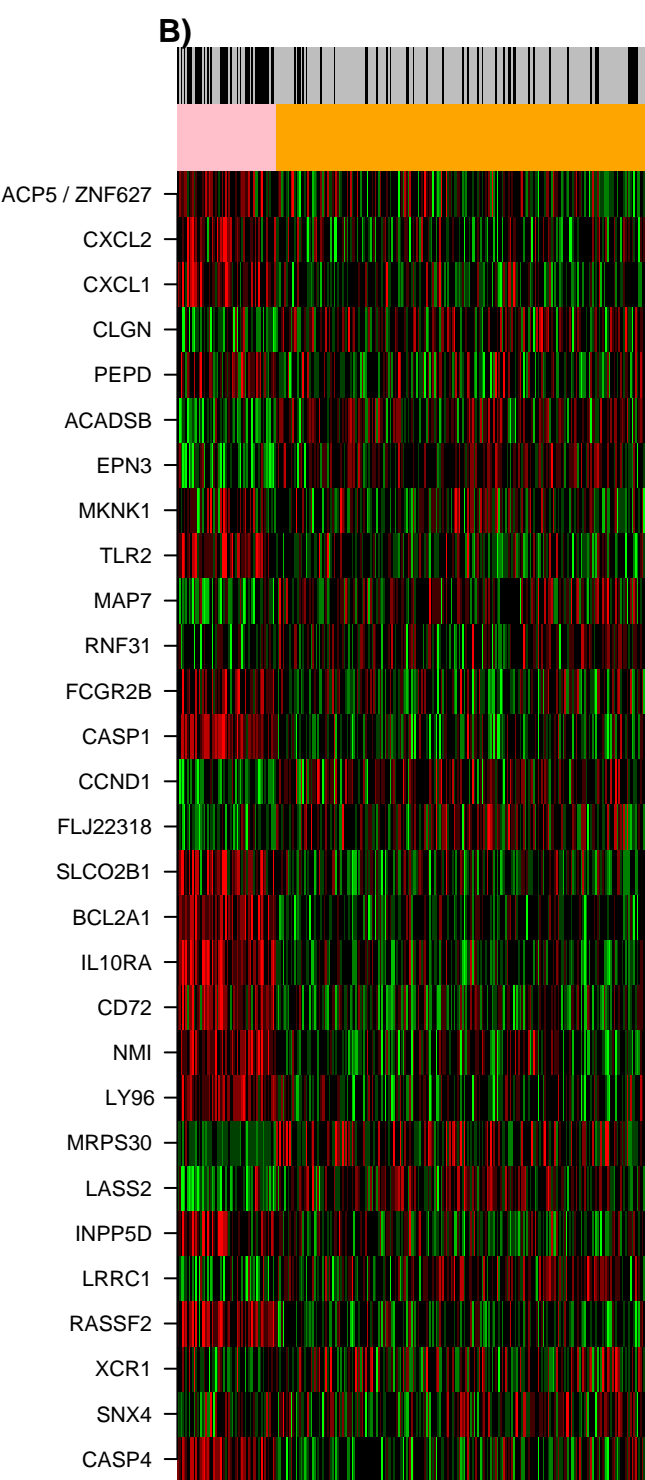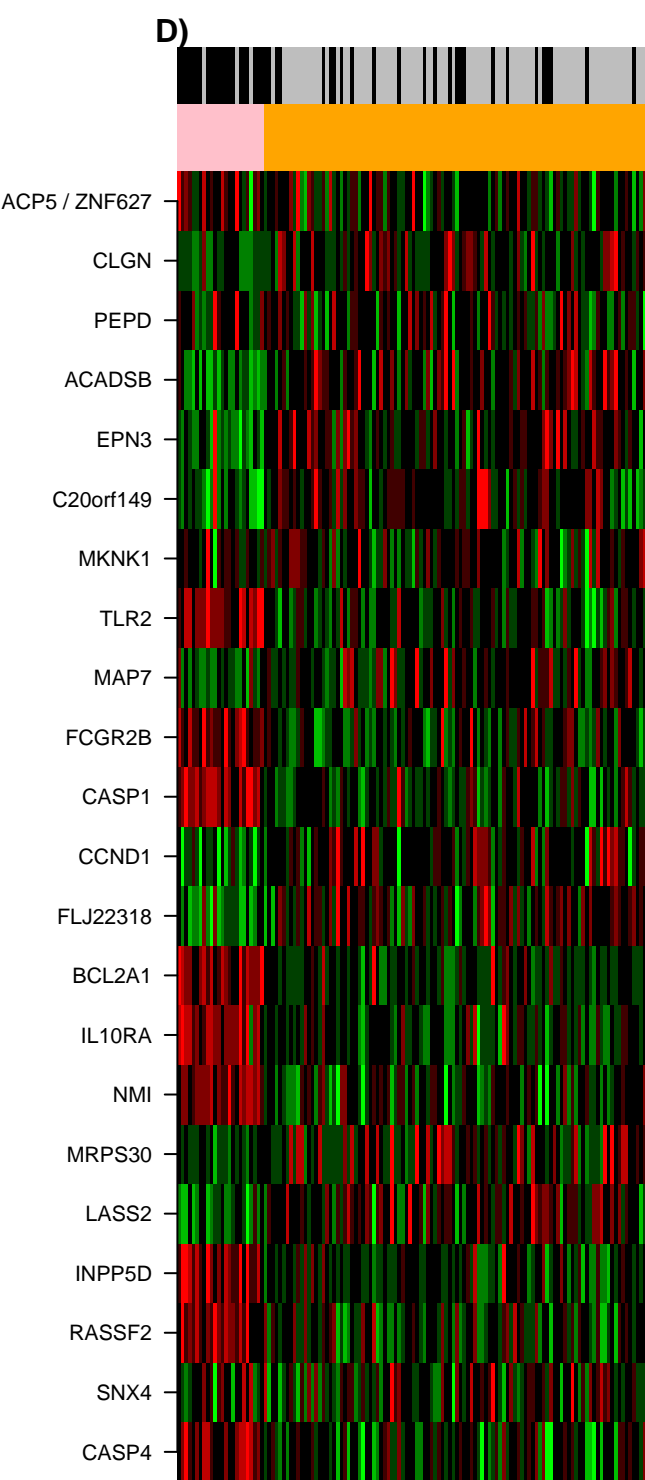

Supplement: Additional data file 6 — The expression classifier for the low-GII subgroup in four independent external breast cancer cohorts: A, van de Vijver et al. [35]; B, Wang et al. [34]; C, Sotiriou et al. [36]; and D, CAL [6]. Samples in the predicted putative low-GII subgroup are labeled in pink, the rest of the samples are shown in orange. The top color bar denotes ER status (black, ER-; gray, ER+). In the heatmaps, red denotes relative overexpression and green denotes relative underexpression. [file gb-2007-8-10-r215-S6.pdf]

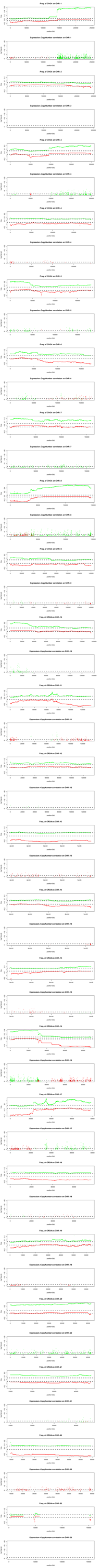

Supplement: Additional data file 7 — For each chromosome we plot (i) the frequency of gain (green) and loss (red) profiles of CRA over the 171 tumors, and (ii) the p values (log10 scale) of Agilent probes in these regions that evaluate the association between copy number gain and overexpression (green), or loss and underexpression (red). Threshold lines of 5% gain and 5% loss and 0.05 significance level are also shown (black). [file gb-2007-8-10-r215-S7.pdf]

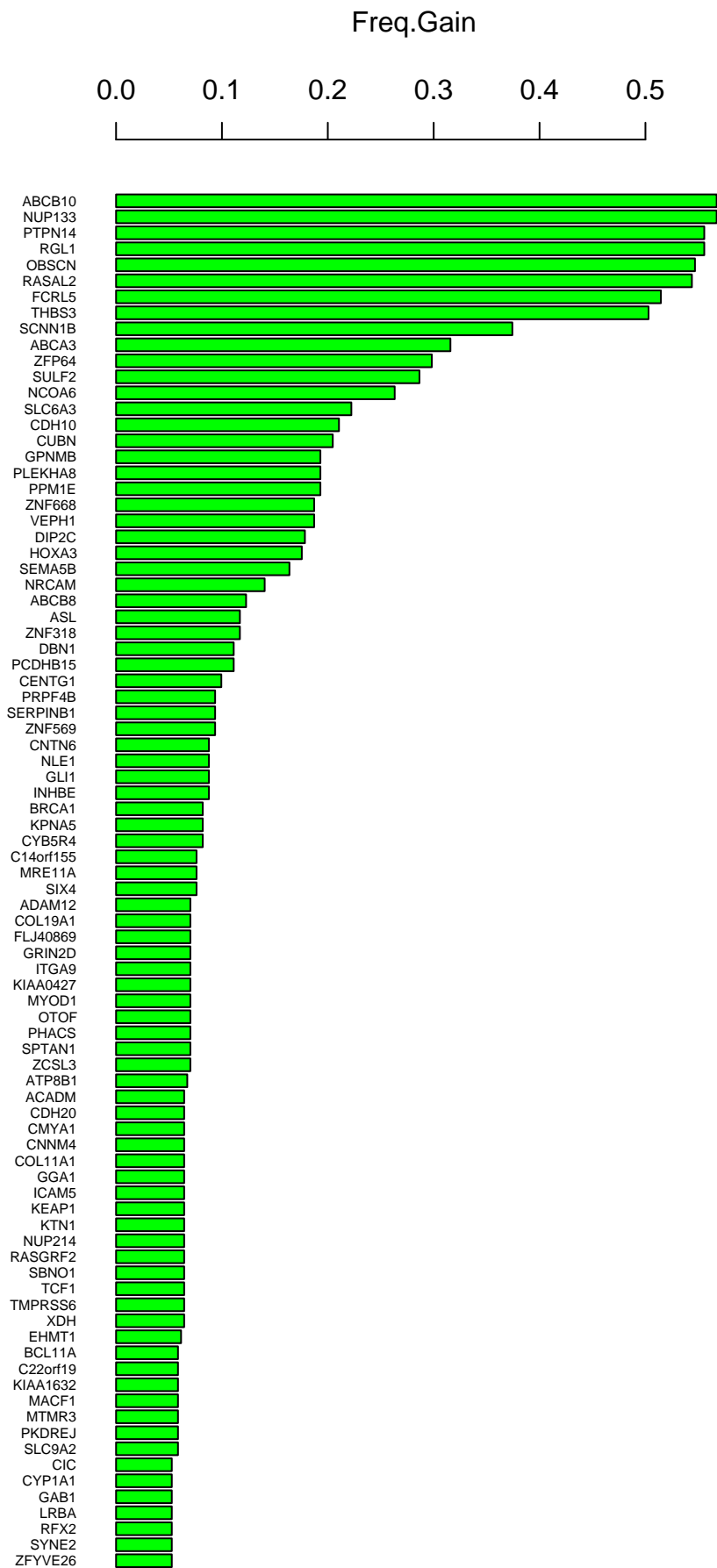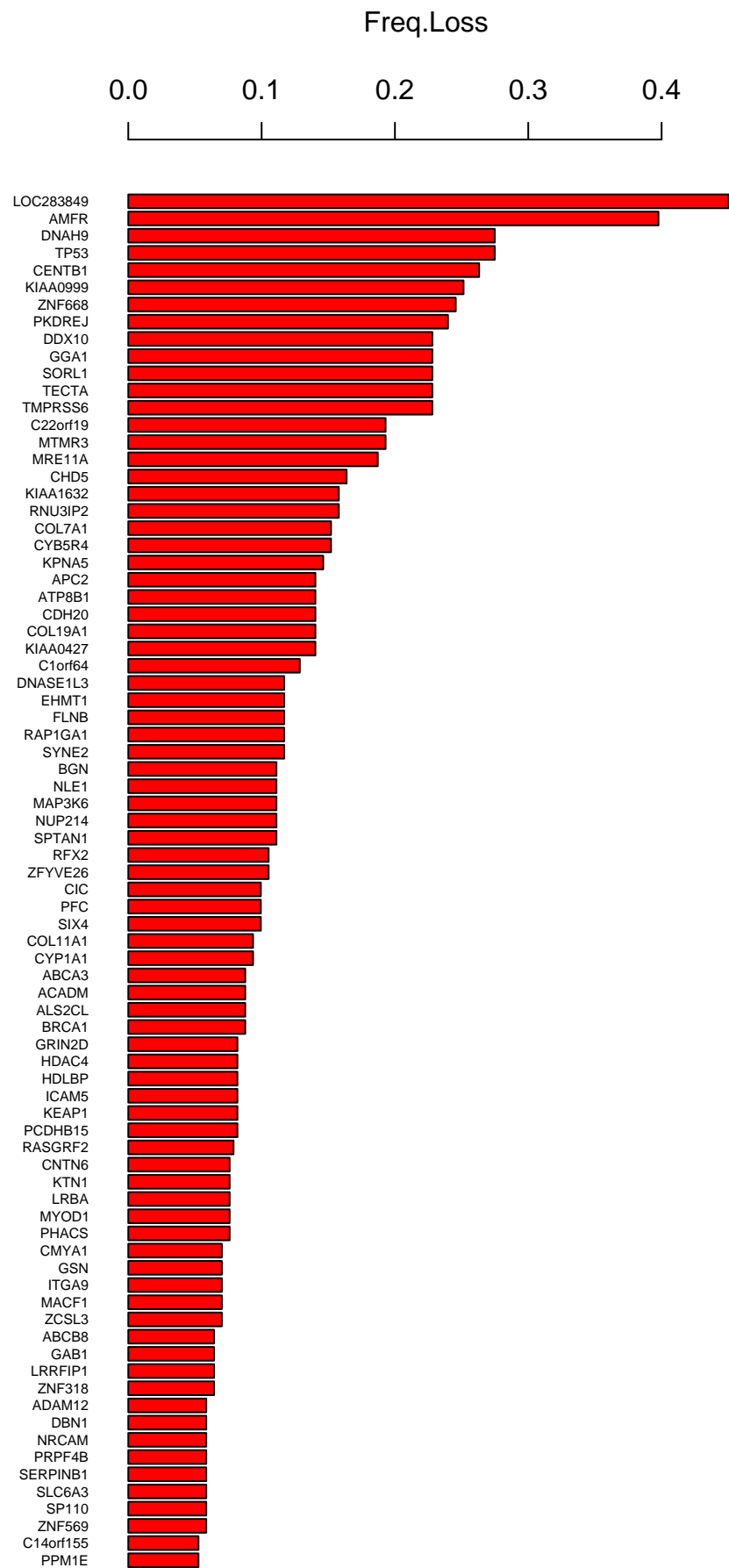

Supplement: Additional data file 10 — Frequency of gains (green) and loss (red) for the most frequently altered CAN genes. [file gb-2007-8-10-r215-S10.pdf]

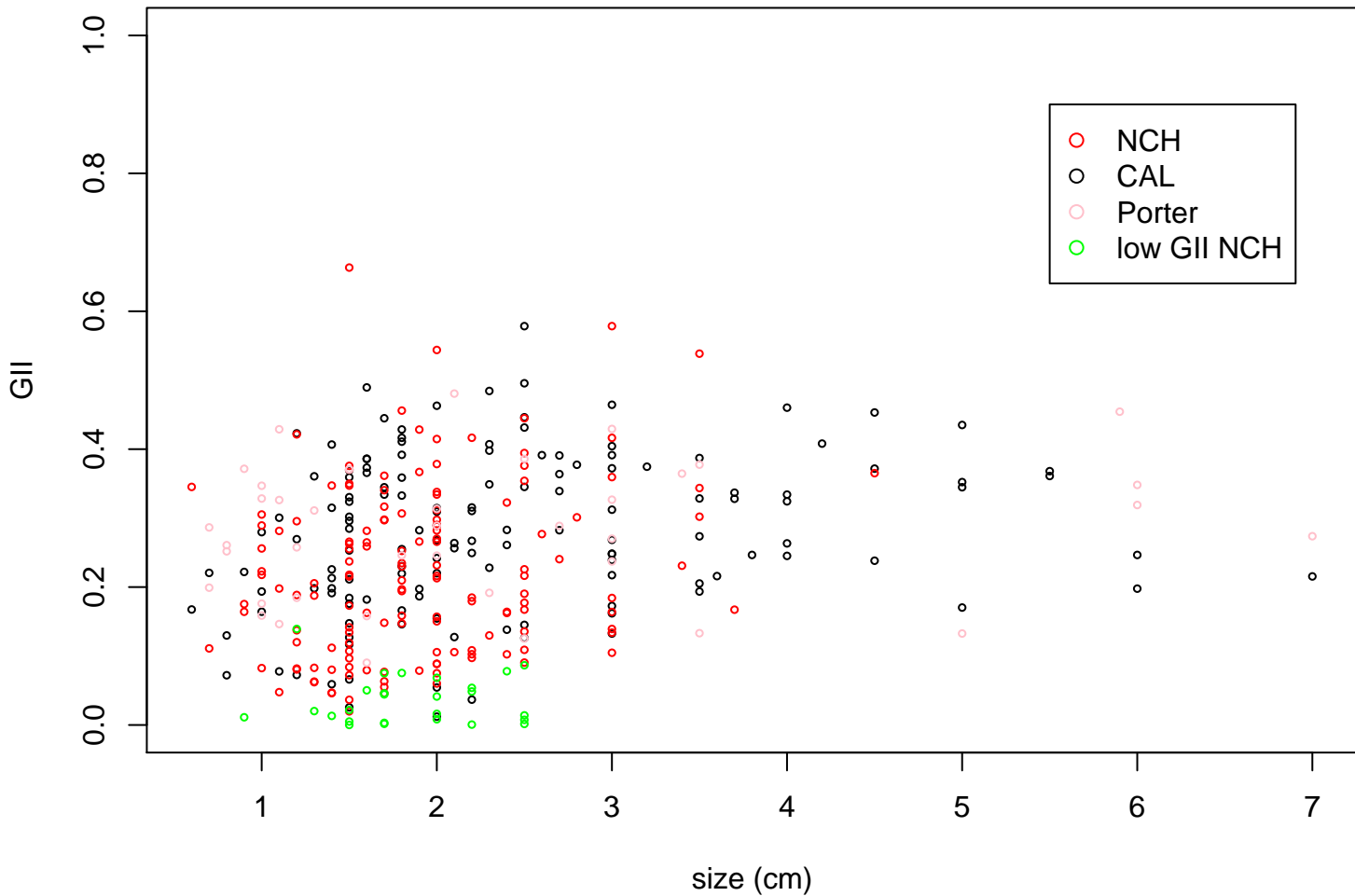

Supplement: Additional data file 12 — GII is plotted against tumor size for the Nottingham City Hospital cohort profiled in this study (NCH) (red), the cohort from California (CAL) profiled in [6] (black) and the cohort (Porter) profiled in [11] (pink). Samples in the NCH cohort that clustered in the 26-sample low-GII subgroup are shown in green. [file gb-2007-8-10-r215-S12.pdf]
