# Supplementary material for: High-resolution aCGH and expression profiling identifies a novel genomic subtype of ER negative breast cancer
Source: Genome Biol. 2007 Oct 7;8(10):R215. doi: 10.1186/gb-2007-8-10-r215 (PMC2246289; doi:10.1186/gb-2007-8-10-r215)
Supplement: Additional data file 4 — Cluster stability analysis of the hierarchical clustering over the 1063 merged regions and 171 breast tumor samples, using the R-package pvclust. The red numbers indicate the robustness index of the clusters. There was only one cluster with a robustness index larger than 90% and containing more than 20 samples. [file gb-2007-8-10-r215-S4.pdf]

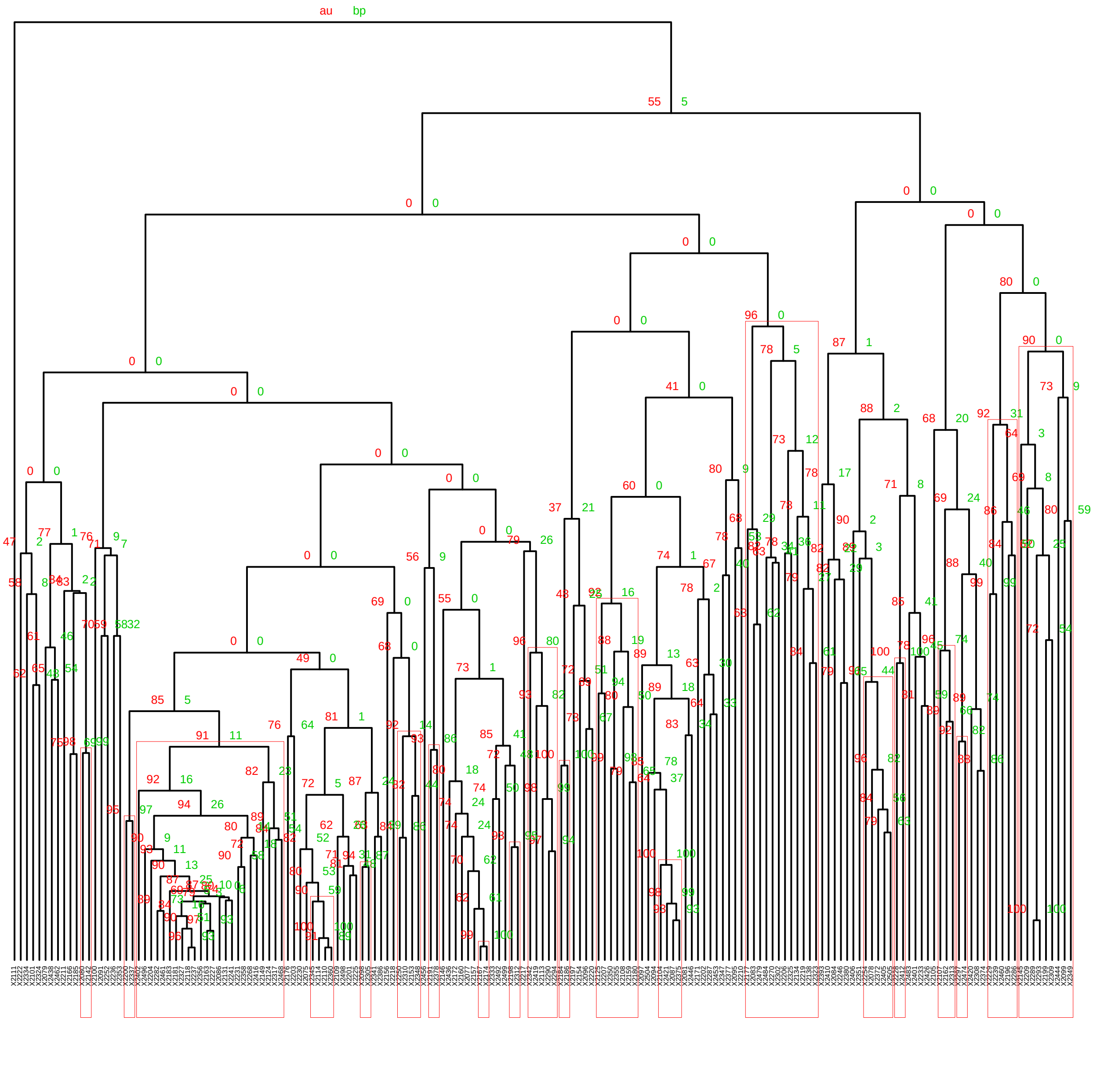

Cluster method: complete
