# Supplementary material for: High-resolution aCGH and expression profiling identifies a novel genomic subtype of ER negative breast cancer
Source: Genome Biol. 2007 Oct 7;8(10):R215. doi: 10.1186/gb-2007-8-10-r215 (PMC2246289; doi:10.1186/gb-2007-8-10-r215)
Supplement: Additional data file 11 — Tables of CAN genes, 'kinome' genes, 'phosphatome' genes and 'chromatinome' genes frequently altered across breast tumors and also showing coordinate aberrant expression. [file gb-2007-8-10-r215-S11.pdf]

Supplementary File S1 - Tables of alteration for selected gene sets and gene families

CAN-genes

Breast cancer CAN-genes with significant gains or losses and showing significant up or downregulation of expression. Columns label the chromosome (CHR), the frequency of gain and loss (Freq.Gain & Freq.Loss), whether the expression association (Expr) is between copy-number gain and overexpression (G) or copy-number loss and underexpression (L), and the p-value of significance as determined by the Wilcoxon rank sum test (pval).

Name	CHR	Freq.Gain	Freq.Loss	Expr	pval
ABCB10	1	0.56725	0.0117	G	$< 10^{-4}$
NCOA6	20	0.26316	0.00585	G	$< 10^{-4}$
NUP133	1	0.56725	0.0117	G	$< 10^{-4}$
OBSCN	1	0.54678	0.0117	G	$< 10^{-4}$
PPM1E	17	0.19298	0.05263	G	0.06
C22orf19	22	0.05848	0.19298	L	0.0006
DDX10	11	0.04678	0.22807	L	0.001
GGA1	22	0.06433	0.22807	L	0.06
SORL1	11	0.04094	0.22807	L	0.04

The phosphatome

Phosphatases with significant gains or losses and showing significant up or downregulation of expression. Columns label the chromosome (CHR), the frequency of gain and loss (Freq.Gain & Freq.Loss), whether the expression association (Expr) is between copy-number gain and overexpression (G) or copy-number loss and underxpression (L), and the p-value of significance as determined by the Wilcoxon rank sum test (pval).

Name	CHR	Freq.Gain	Freq.Loss	Expr	pval
PTPRA	20	0.22807	0.02339	G	0.003
PTPRC	1	0.54971	0	G	0.09
DUSP10	1	0.55556	0	G	0.098
DUSP12	1	0.55556	0.00585	G	0.0002
DUSP15	20	0.25146	0.00585	G	0.008
PTP4A3	8	0.45029	0.07018	G	0.08
MTMR4	17	0.18713	0.05263	G	0.06
PTPRK	6	0.09357	0.15497	L	0.02
MTMR2	11	0.07018	0.19883	L	0.02
EYA4	6	0.08772	0.15205	L	0.05

Histones and Chromatin Modifiers

Histones and chromatin modifiers with significant gains or losses and showing significant up or downregulation of expression. Columns label the chromosome (CHR), the frequency of gain and loss (Freq.Gain & Freq.Loss), whether the expression association (Expr) is between copy-number gain and overexpression (G) or copy-number loss and underxpression (L), and the p-value of significance as determined by the Wilcoxon rank sum test (pval). Abbreviations: histone methyltransferase (HMT), helicase (HELIC), histone acetyltransferase (HAT), histone deacetyltransferase (HDAC), histone (HIST), histone demethyltransferase (HDMT), bromodomain associated (BROM.DOM), chromatin assembly (CHROM.ASSEM.), chromatin domain associated (CHROM.DOM).

Name	Type	CHR	Freq.Gain	Freq.Loss	Expr	pval
ARID4B	HDMT	1	0.55166	0.0078	G	$< 10^{-4}$
ADA		20	0.26901	0.01754	G	0.03
ASH2L	HMT	8	0.31579	0.14035	G (L)	$< 10^{-5}$ (0.04)
CHAF1B		21	0.10526	0.08187	G	$< 10^{-4}$
CHD1L / POLR3C	HELIC	1	0.49269	0.00731	G	0.02
CHRAC1		8	0.50292	0.04094	G	0.08
CREBBP	HAT	16	0.33333	0.07018	G	0.03
DNMT3B		20	0.24561	0.0117	G	0.006
FBXO18	HELIC	10	0.20468	0.01754	G	0.05
FKBP1A		20	0.23392	0.02339	G	0.08
H2AFV		7	0.12865	0.04094	G	0.006
H3F3B		17	0.16959	0.11111	G	$< 10^{-5}$
HDAC2	HDAC	6	0.09357	0.1462	G (L)	0.0001 (0.04)
HIST1H1B	HIST	6	0.12865	0.02924	G	0.009
HIST1H1D	HIST	6	0.1345	0.02924	G	0.07
HIST1H2BD	HIST	6	0.1345	0.02924	G	0.09
HIST1H2BE	HIST	6	0.1345	0.02924	G	0.01
HIST1H2BI	HIST	6	0.1345	0.02339	G	0.05
HIST1H3C	HIST	6	0.1345	0.02924	G	0.04
HIST2H2AA	HIST	1	0.52047	0.00585	G	< 0.1
HIST2H4	HIST	1	0.51901	0.01901	G	$< 10^{-4}$
JARID1B / CYB5R1	HDMT	1	0.57018	0.00877	G	0.0003
JMJD1B	HDMT	5	0.10526	0.07602	G	0.06
L3MBTL		20	0.26316	0.02924	G	0.005
MYST1	HAT	16	0.19883	0.20468	G	0.05
PARP1		1	0.55556	0	G	$< 10^{-5}$
RBBP6		16	0.36842	0.03509	G	0.03
SETDB1	HMT	1	0.53216	0.0117	G	0.003
SMYD3	HMT	1	0.5731	0.01754	G	0.09
SUV39H2	HMT	10	0.21053	0.01754	G	$< 10^{-5}$
THEX1		8	0.1345	0.24561	G	0.08
THRAP4		17	0.16374	0.07018	G	0.0001
UBE2D2		5	0.11111	0.07602	G	0.01
UBE2V2		8	0.35673	0.04094	G	$< 10^{-4}$
WHSC1L1	HMT	8	0.32164	0.12865	G	0.0007
BRD1	BROM.DOM	22	0.05848	0.23392	L	0.04
CHAF1A	CHROM.ASSEM.	19	0.04094	0.11696	L	0.05
CHD9	CHROM.DOM	16	0.07018	0.34795	L	0.01
EP300	HAT	22	0.06433	0.22807	L	0.03
H1F0	HIST	22	0.07018	0.22807	L	0.03
MBD2		18	0.07602	0.14035	L	0.0004
MIZF	MBD2-interact.	11	0.03509	0.24561	L	0.005
MLL	HMT	11	0.04094	0.23977	L	0.09
NCOR1 / TTC19	HDAC-complex	17	0.02924	0.25146	L	$< 10^{-4}$
NHP2L1		22	0.05848	0.22807	L	0.01
PRMT7	HMT	16	0.02924	0.4386	L	0.0002
SAP18	HDAC-complex	13	0.05263	0.16959	L	0.0004
SIRT3	HDAC	11	0.02924	0.15205	L	0.002
SLBP		4	0.07018	0.14035	L	0.07
UBE2G1		17	0.04094	0.2807	L	0.002
UBE2L3		22	0.07018	0.21637	L	0.002

The Kinome

Kinases with significant gains or losses and showing significant up or downregulation of expression. Columns label the chromosome (CHR), the frequency of gain and loss (Freq.Gain & Freq.Loss), whether the expression association (Expr) is between copy-number gain and overexpression (G) or copy-number loss and underxpression (L), and the p-value of significance as determined by the Wilcoxon rank sum test (pval).

Name	CHR	Freq.Gain	Freq.Loss	Expr	pval
ATR	3	0.18713	0.02339	G	0.002
CDK5	7	0.12281	0.06433	G	0.09
CLK2	1	0.50292	0.02924	G	$< 10^{-4}$
HER2/ERBB2	17	0.27485	0.07018	G	$< 10^{-5}$
GSK3B	3	0.16959	0.02339	G	0.08
LIMK1	7	0.15789	0.05263	G	0.03
MARK1	1	0.55556	0	G	0.08
PHKG1	7	0.1345	0.02339	G	0.04
TRIO	5	0.21053	0.02924	G	0.02
DYRK3	1	0.5731	0	G	0.06
RIPK2	8	0.48538	0.01754	G	$< 10^{-4}$
MAPKAPK2	1	0.5731	0	G	0.008
SGK2	20	0.26316	0.02924	G	0.005
BCKDK	16	0.19298	0.20468	G	0.07
TLK2	17	0.23977	0.03509	G	0.02
ZC2/TNIK	3	0.15497	0.02924	G	0.003
SGK3	8	0.42105	0.01754	G	0.04
PIK3R4	3	0.15789	0.01754	G	0.01
CDKL3	5	0.11111	0.10526	G	0.001
PBK	8	0.12865	0.27485	G	0.01
SCYL3	1	0.54971	0.00585	G	$< 10^{-4}$
OBSCN	1	0.54678	0.0117	G	$< 10^{-4}$
NEK7	1	0.54386	0.00585	G	0.09
ILK	11	0.02924	0.12865	L	0.03
NEK3	13	0.02924	0.21053	L	0.002
MAP2K4	17	0.02339	0.26901	L	0.0001
TESK1	9	0.08772	0.10526	L	0.004
RIOK3	18	0.07602	0.12281	L	0.07
STK33	11	0.02924	0.12281	L	0.04
MLKL	16	0.04094	0.4386	L	0.005
PIM3	22	0.05848	0.24561	L	0.03
ULK3	15	0.05848	0.09357	L	0.06
